# Supplementary material for: An in Silico Approach to Identifying TF Binding Sites: Analysis of the Regulatory Regions of BUSCO Genes from Fungal Species in the Ceratocystidaceae Family
Source: Genes (Basel). 2023 Mar 31;14(4):848. doi: 10.3390/genes14040848 (PMC10137650; doi:10.3390/genes14040848)

Supplementary information

Table S1: The BUSCO genes used in this study. The table below shows for each of the 20 selected genes, their gene names, GenBank accession numbers, BUSCO IDs as well as a brief description of the biological function of their protein products.

Key	Gene name	accession	BUSCO ID	Function
	Dynein heavy chain	KAF7735571.1	EOG092D0072	Serves as molecular motor, facilitate directed movement along microtubules. Dynein heavy chains are responsible for converting chemical energy into mechanical energy which is applied to the microtubule surface.
	Pre-mRNA-processing-splicing factor 8	KAF7735910.1	EOG092D00LL	Serves as pre-mRNA spicing factor, PRPF8 plays an important role in spliceosome assembly.
	General negative regulator of transcription subunit 1	KAF7732669.1	EOG092D0124	Negative regulation of transcription specifically negatively regulates the use of the TATA box element.
	SNF2-related protein	KAF7739295.1	EOG092D01IY	Facilitate chromatin remodelling by applying tortional strain to DNA and promoting nucleosome movement.
	U3 snoRNP protein	KAF3316205.1	EOG092D01J4	Facilitates 18S rRNA processing and small subunit ribosome formation in eukaryotes.
	Phospholipase D family protein	KAF7734797.1	EOG092D01MX	Catalyses the hydrolysis of the phosphodiester bond of glycerophospholipids to generate phosphatidic acid and a free headgroup.
	Cell morphogenesis protein PAG1	KAF7733949.1	EOG092D01QP	Facilitates normal morphogenesis in vegetatively growing cells.
	phosphatidylinositol-4- kinase	KAF3315303.1	EOG092D01WX	Phosphatidylinositol 4-kinases (PI4Ks) synthesize phosphatidylinositol 4-phosphate (PI4P). PI4P is a key lipid for the identity of the Golgi and trans-Golgi network (TGN).

	Clathrin, heavy chain	KAF7737936.1	EOG092D01YA	Clathrin is a key structural protein that forms a lattice-like complex that includes two heavy chain subunits (CHC1 and CHC2) and two light chain subunits (CLC1 and CLC2).
	E3 ubiquitin-protein ligase listerin	KAF7734492.1	EOG092D01ZK	Listerin E3 Ubiquitin Protein Ligase 1 encoded protein is a part of the RQC complex (The complex plays a role in the degradation of the polybasic-mediated stalled protein).
	Transcriptional regulatory protein sin3	KAF3313021.1	EOG092D02YC	Corepressor that facilitates transcriptional silencing via a complex with associated histone deacetylases (HDACs). The core Sin3–HDAC complex interacts with a wide variety of repressors and corepressors.
	UDP-glucose:glycoprotein glucosyltransferase	KAF7736011.1	EOG092D03RC	UDP-glucose: glycoprotein glucosyltransferase (UGGT) plays a significant role in the quality control mechanism that newly synthesized glycoproteins undergo in the endoplasmic reticulum.
	Sister chromatid cohesion protein 2	KAF3317641.1	EOG092D03RY	Essential for the biorientation of chromosomes on the mitotic or meiotic spindle, and thus responsible for chromosome segregation.
	DNA repair protein rad50	KAF3314195.1	EOG092D042R	Forms part of a complex responsible for DNA double-strand break repair to recognize and process DNA ends as well as signal for cell cycle arrest.
	Anaphase-promoting complex subunit 1	KAF3311063.1	EOG092D0454	Forms part of the Anaphase-promoting complex which mainly required to induce progression and exit from mitosis by inducing proteolysis of different cell cycle regulators.
	THO complex subunit 2	KAF3316359.1	EOG092D0564	Forms part of the THO complex is a key component in the co-transcriptional formation of messenger ribonucleoparticles that are competent to be exported from the nucleus.
	PAN2-PAN3 deadenylation complex catalytic subunit PAN2	KAF7734681.1	EOG092D05RI	Is the catalytic subunit of the poly(A)-nuclease (PAN) deadenylation complex and is a deadenylase involved in general and miRNA-mediated mRNA turnover.
	Transcription elongation factor spt6	KAE8445330.1	EOG092D05X9	Coordinates nucleosome disassembly and reassembly, transcriptional elongation, and mRNA processing.

	Elongation factor EF-Tu	KAF7737480.1	EOG092D0ACX	Catalyses the binding of aminoacyl-tRNA to the A-site of the ribosome inside living cells.
	MIFG and Upf2 domain-containing protein	KAF7733737.1	EOG092D0AI2	Involved in detecting and degradation of mRNAs with premature stop codons

Key:

- Protein turnover
- Cell division
- Transcription and mRNA processing
- Transcription and rRNA processing
- Intracellular vesicle processing and trafficking
- DNA repair
- Chromatin remodelling
- Cell signalling
- Cytoskeletal transport

Table S2: The highest p-value per motif. For each gene, the table below shows the motif number and the highest p-value for each motif.

	Highest P-value	Motif number
**EOG092D02YC**		
	1.78E-17	1
	4.70E-12	2
**EOG092D00LL**		
	1.57E-14	1
	2.61E-16	2
	1.03E-13	3
	2.40E-12	4
	3.02E-9	5
	9.13E-13	6
**EOG092D03RC**		
	1.9E-9	6
	6.22E-8	9
	3.97E-7	14
**EOG092D03RY**		
	9.55E-13	2
	1.8E-9	5
	9.56E-7	18
**EOG092D0454**		
	5.12E-6	22
**EOG092D0564**		
	2.04E-9	2
	1.37E-9	3
	1.62E-10	4
**EOG092D0124**		
	3.01E-9	1
	1.18E-9	12
	8.47E-6	20
**EOG092D01IY**		
	7.68E-10	4
	1.84E-6	9
**EOG092D01MX**		
	1.14E-5	28
**EOG092D01WX**		
	1.85E-24	1
	4.09E-25	2
	9.91E-19	4
	1.01E-18	6

	1.27E-10	7
	1.96E-8	9
**EOG092D01YA**		
	6.29E-16	1
	2.16E-11	2
	4.54E-10	3
	3.32E-11	5
	3.06E-8	8
	5.03E-8	9
	1.85E-6	18
**EOG092D01J4**		
	1.00E-21	1
	3.37E-20	2
	7.17E-20	3
	1.77E-19	4
	4.14E-20	5
	1.62E-15	6
	3.54E-12	7
	1.08E-7	9
	4.85E-8	12
	7.26E-8	13
**EOG092D0072**		
	9.26E-9	1
	1.40E-7	4
**EOG092D05X9**		
	2.00E-11	1
	3.10E-7	15
**EOG092D01ZK**		
	5.22E-9	2
	1.10E-7	3
**EOG092D0ACX**		
	1.00E-15	1
	3.45E-17	2
	2.26E-20	3
	2.00E-11	4
	1.37E-13	5
	2.47E-12	6
	1.01E-12	8
	1.9E-10	11
	3.00E-13	18
	1.15E-09	20
**EOG092D0AI2**		
	1.6E-14	1
	1.00E-18	2

	3.47E-20	6
	2.00E-11	7
	2.55E-15	8
	2.01E-12	20

Table S3: Number of identified motifs per gene. For each gene in the table below, the number of motifs that met cut off parameters, the sequence tags of each of these motifs, the number of genomes that have these motifs (Sites), the motif lengths, and position of the motifs relative to the ATG is shown.

Motif number	Sequence tag	E-value	Sites	Length	Position from ATG
	EOG092D02YC				
1	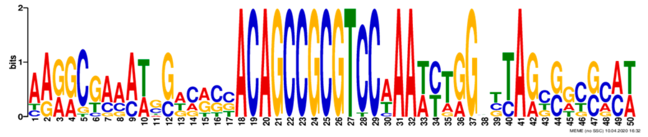	1.4E-199	20	50	373-444
2	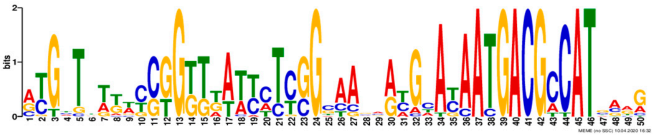	1.5E-133	20	50	468-535
	EOG092D00LL				
1	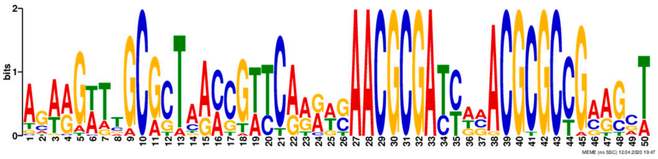	9.3E-265	24	50	43-95
2	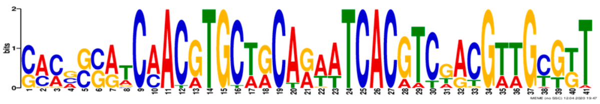	6.9E-215	22	41	203-256
3	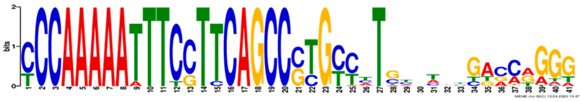	1.0E-214	24	41	287-339
4	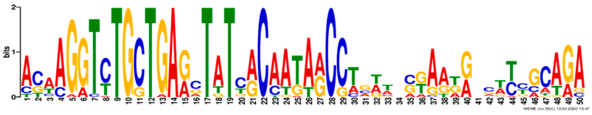	1.3E-169	23	50	139-673
5	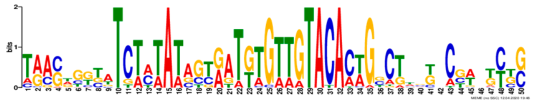	1.4E-148	24	50	514-896
6	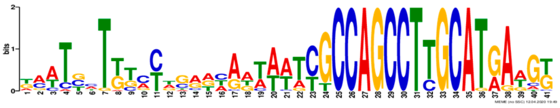	5.9E-132	22	41	245-297
	EOG092D03RC				

6	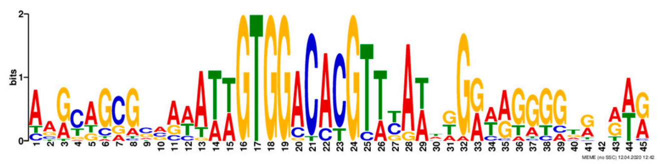	2.2E-095	20	45	124-791
9	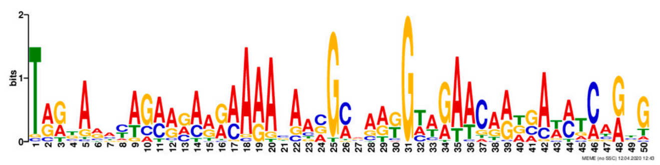	6.3E-072	24	50	20-944
14	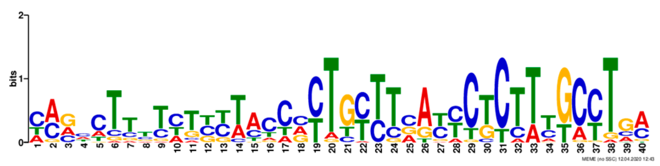	1.2E-041	24	40	45-841
	EOG092D03RY				
2	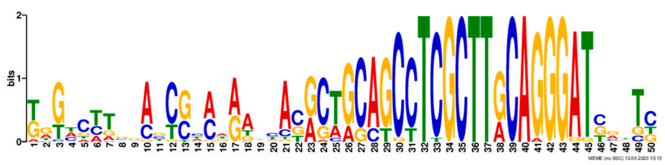	2.3E-183	24	50	499-948
5	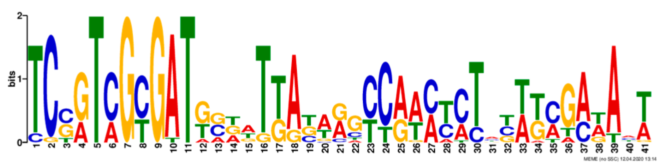	7.0E-088	20	41	23-954
18	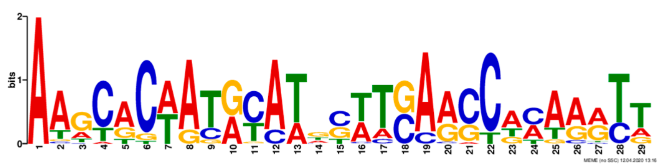	1.5E-043	23	29	138-807
	EOG092D0454				
22	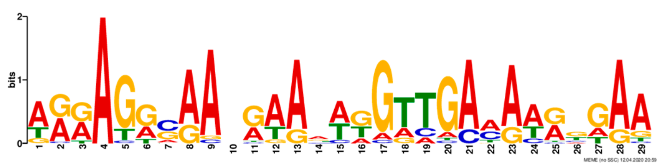	4.1E-024	23	29	2-950
	EOG092D0564				
2	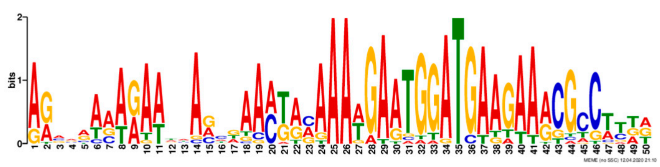	1.2E-119	21	50	154-632
3	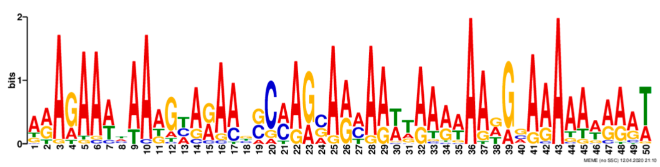	1.4E-104	22	50	196-693

4	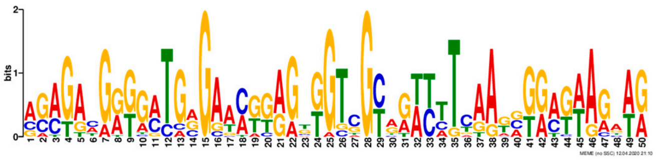	7.3E-103	20	50	4-741
	EOG092D0124				
1	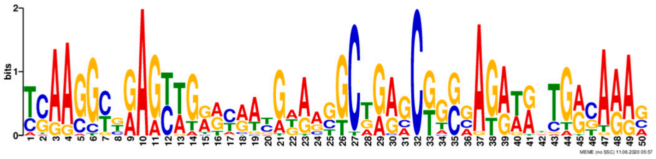	3.6E-155	25	50	17-796
12	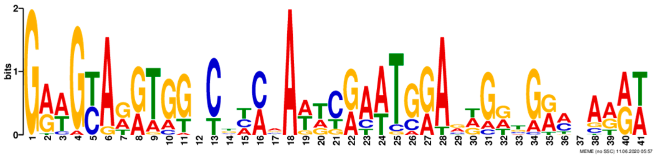	1.1E-066	22	50	117-637
20	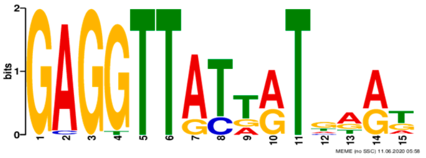	1.3E-030	25	15	1-898
	EOG092D01IY				
4	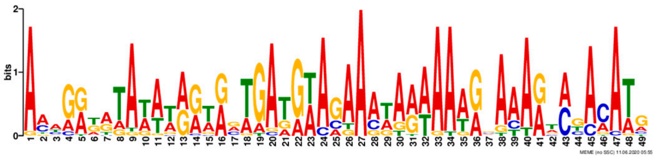	3.2E-096	22	50	205-900
9	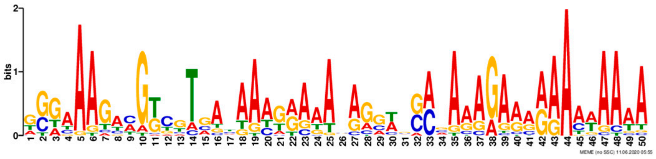	3.7E-070	24	50	299-906
	EOG092D01MX				
28	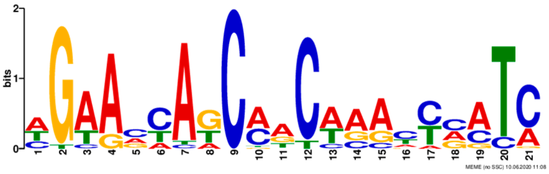	3.6E-012	25	21	113-906
	EOG092D01WX				
1	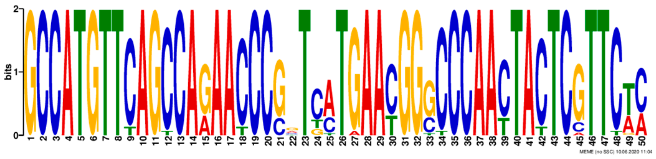	4.0E-359	21	50	516-829

2	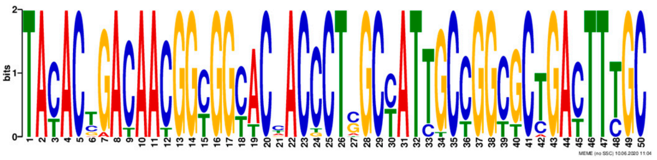	9.7E-322	20	50	673-943
4	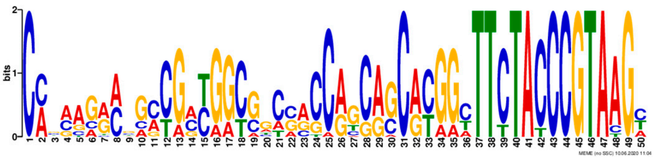	4.5E-186	21	50	573-889
6	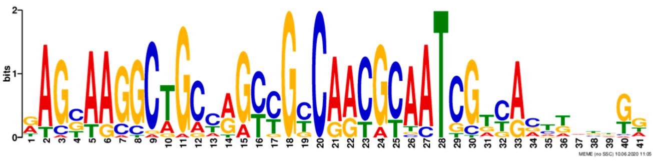	1.6E-131	25	41	17-921
7	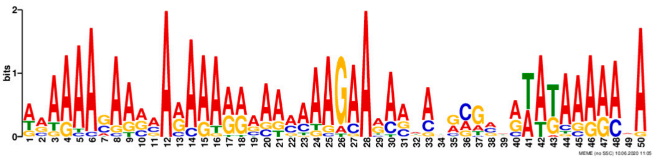	4.7E-103	21	50	15-385
9	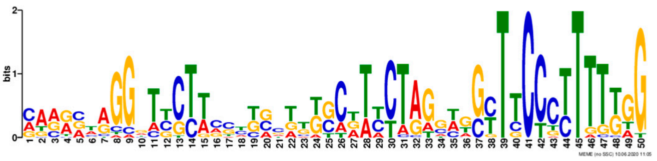	7.1E-079	22	50	200-462
	EOG092D01YA				
1	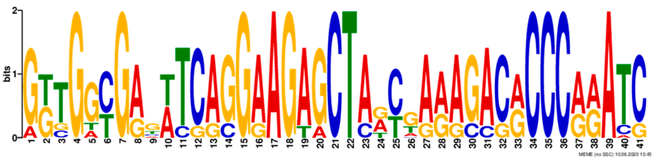	5.6E-210	22	41	16-255
2	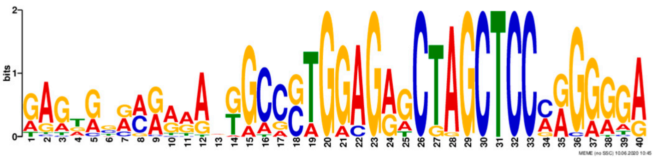	2.4E-169	24	40	39-204
3	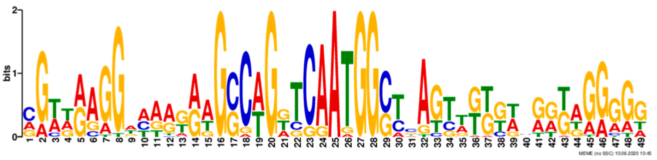	3.3E-152	25	49	152-493
5	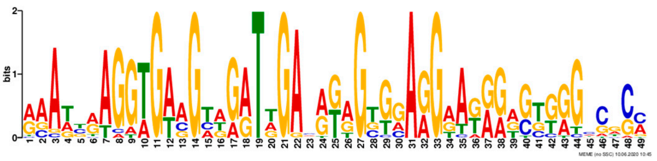	6.7E-114	20	49	261-552

8	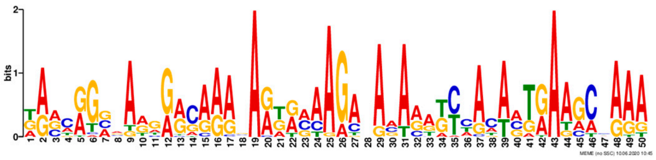	5.8E-098	25	50	143-908
9	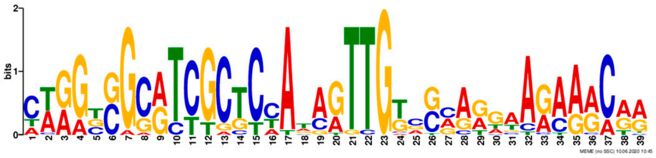	1.1E-075	20	39	64-873
18	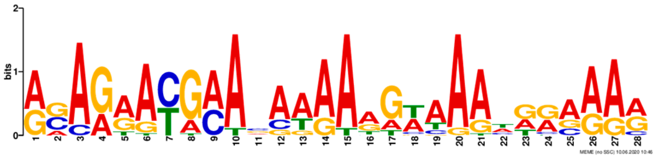	7.4E-037	25	28	67-972
	EOG092D01J4				
1	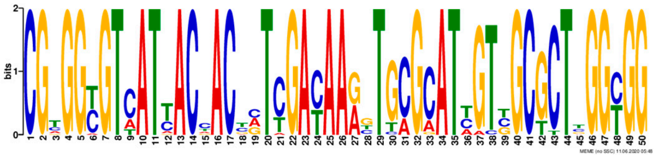	1.4E-335	23	50	758-940
2	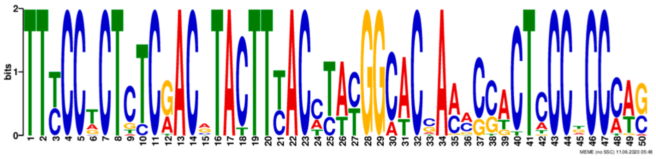	2.7E-311	23	50	727-829
3	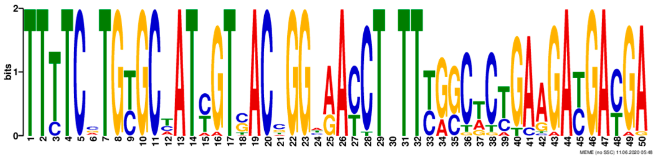	6.6E-316	23	50	563-742
4	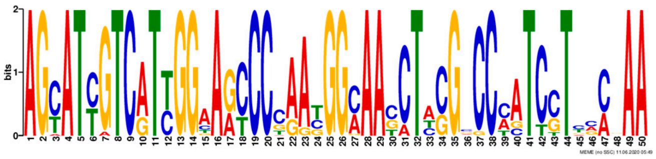	1.7E-298	23	50	485-664
5	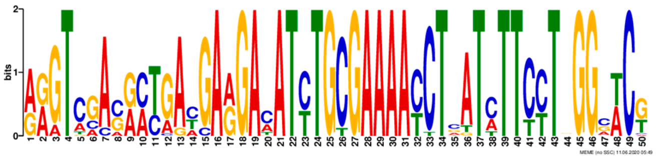	4.4E-294	23	50	708-890
6	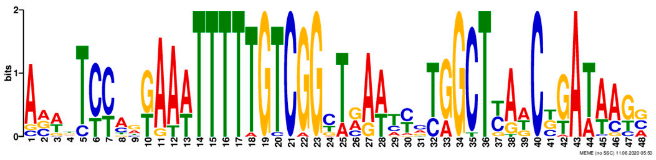	9.5E-201	23	48	113-331

7	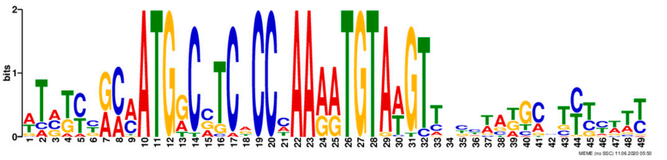	3.1E-143	23	49	411-571
9	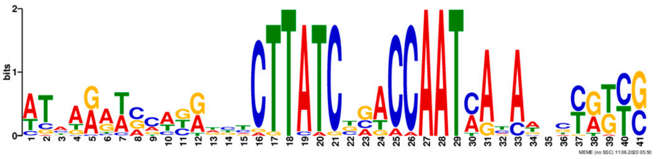	4.9E-102	25	41	79-758
12	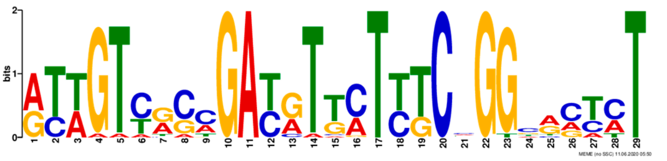	1.9E-091	24	29	111-796
13	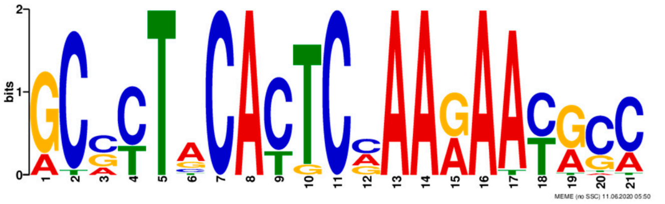	7.9E-081	24	21	542-883
	EOG092D0072				
1	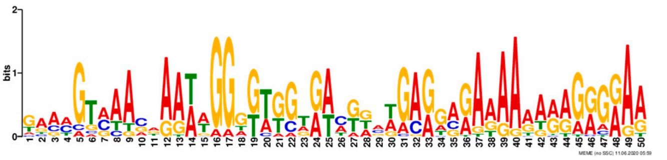	7.2E-097	24	50	251-685
4	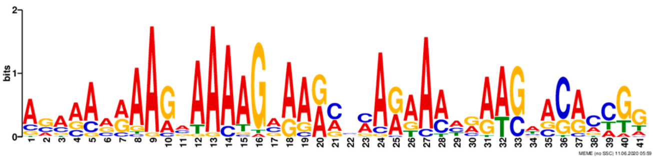	9.7E-067	24	41	169-952
	EOG092D05X9				
1	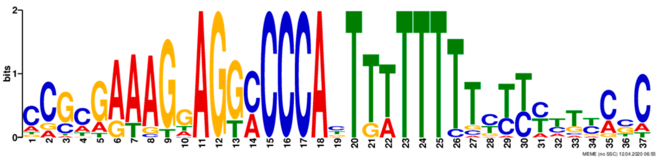	1.0E-128	22	37	277-448
15	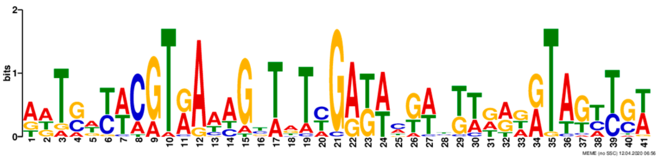	1.8E-041	20	41	21-627
	EOG092D01ZK				

2	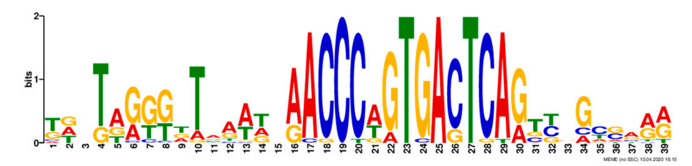	1.6E-102	24	39	556-960
3	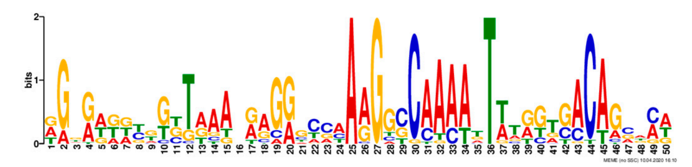	2.0E-089	24	50	238-885
EOG092D0ACX					
1	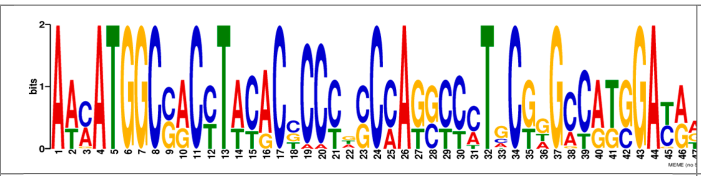	4.5E-240	25	50	926-747
2	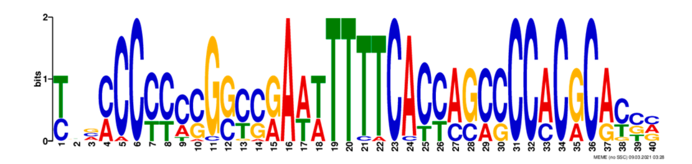	8.4E-164	25	40	719-585
3	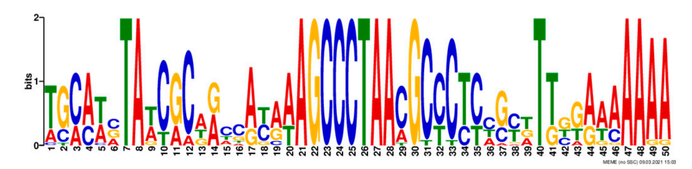	1.7E-170	23	50	352-406
4	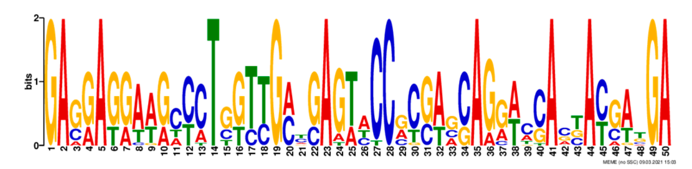	2.1E-169	24	50	538-946
5	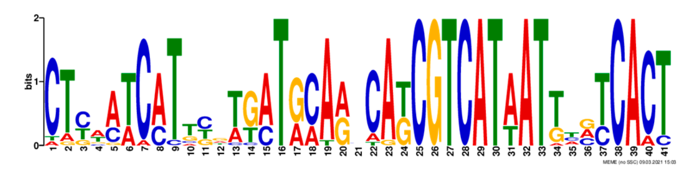	1.1E-129	23	41	168-257
6	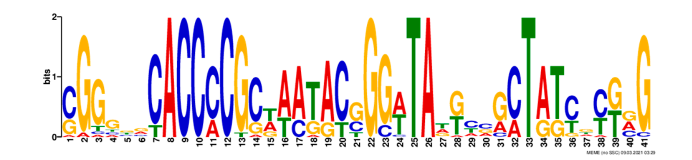	6.6E-128	25	41	823-286
8	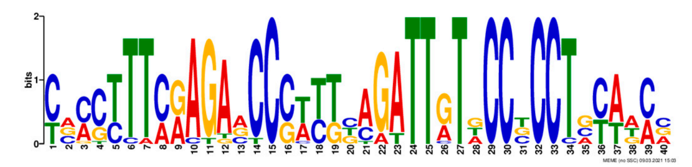	2.2E-098	21	40	434-785

11	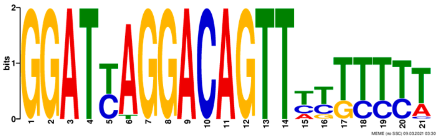	3.2E-088	25	21	757-631
18	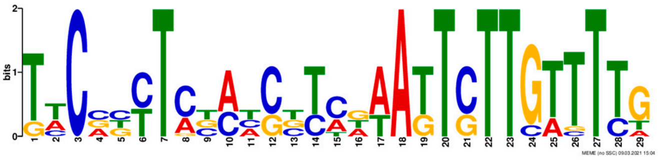	1.5E-044	21	29	11-734
20	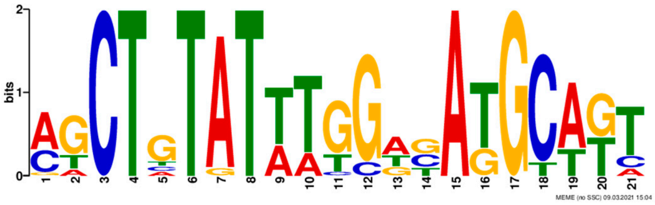	1.1E-034	22	21	155-673
	EOG092D0AI2				
1	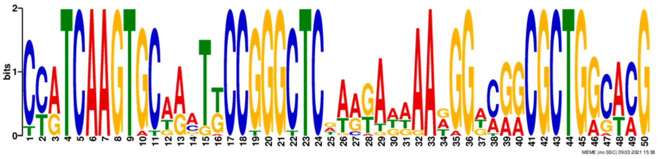	1.9E-247	25	50	162-348
2	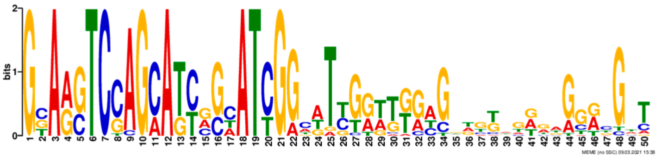	4.6E-104	25	50	244-398
6	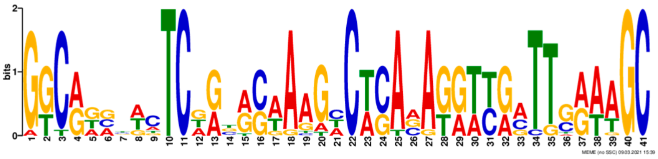	7.5E-074	21	41	113-906
7	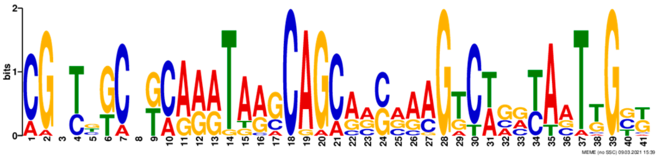	5.2E-066	21	41	173-781
8	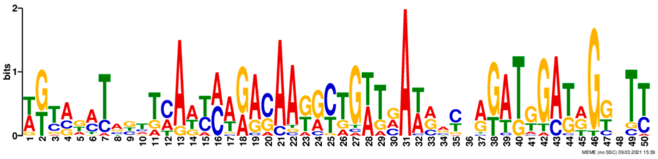	2.1E-057	24	50	58-775
20	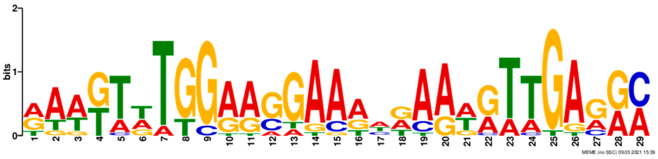	3.4E-025	23	29	58-895
	EOG092D01QP = None met cut off				

	EOG092D042R = None met cut off
	EOG092D05RI = None met cut off

Table S4: Discovered motifs that matched to known TFs

The motif comparison results for both the JASPAR CORE 2018 database and the JASPAR CORE fungi 2018 subcollection. The table below show, the BUSCO IDs of genes that returned matches to either of the two databases, the motifs from each gene that matched to known TFs, names of these known TFs, the protein class of matched TFs and lastly the species of origin of the known TFs.

		JASPAR CORE 2018			JASPAR CORE FUNGI 2018		
Gene ID	Motifs	Matched TFs	Class	species of origin	Matched TFs	Class	species of origin
EOG092D00LL	Motif 1	MBP1::SW16 (MA0330.1)	APSES-type DNA-binding domain	*Saccharomyces cerevisiae*			
		STP3	C2H2 zinc finger factors	*Saccharomyces cerevisiae*			
		RSC3	C6 zinc cluster factors	*Saccharomyces cerevisiae*			
	Motif 2				PHO4	Basic helix-loop-helix factors (bHLH)	*Saccharomyces cerevisiae*
					TYE7 (MA0409.1)	Basic helix-loop-helix factors (bHLH)	*Saccharomyces cerevisiae*
	Motif 3				SUM1 (MA0398.1)	A.T Hook factors	*Saccharomyces cerevisiae*
					YRM1 (MA0438.1)	C6 zinc cluster factors	*Saccharomyces cerevisiae*
	Motif 4				STP4 (MA0397.1)	C2H2 zinc finger factors	*Saccharomyces cerevisiae*
					DOT6 (MA0351.1)	Tryptophan cluster factor	*Saccharomyces cerevisiae*
	Motif 5	-	-	-	-	-	-
	Motif 6	-	-	-	-	-	-
EOG092D01J4	Motif 6	ARF8 (MA0944.1)	B3 domain	*Saccharomyces cerevisiae*	UME6 (MA0412.1)	C6 zinc cluster factors	*Saccharomyces cerevisiae*
		ARF5 (MA0943.1)	B3 domain	*Saccharomyces cerevisiae*	SUM1 (MA0398.1)	A.T Hook factors	*Saccharomyces cerevisiae*
		ARF2 (MA1206.1)	B3 domain	*Saccharomyces cerevisiae*			
		RAP210 (MA1249.1)	AP2/ERF domain	*Saccharomyces cerevisiae*			
		AT3G60490 (MA1223.1)	AP2/ERF domain	*Saccharomyces cerevisiae*			
	Motif 9	GATA3 (MA0037.3)	Other C4 zinc finger-type factors	*Homo sapiens*	DAL80 (MA0289.1)	Other C4 zinc finger-type factors	*Saccharomyces cerevisiae*
		NFYA (MA0060.3)	Other alpha	*Homo sapiens*	HAP3 (MA0314.1)	Heteromeric CCAAT-binding	*Saccharomyces cerevisiae*

					GAT1 (MA0300.1)	Other C4 zinc finger-type factors	*Saccharomyces cerevisiae*
					GLN3 (MA0307.1)	Other C4 zinc finger-type factors	*Saccharomyces cerevisiae*
					GZF3 (MA0309.1)	Other C4 zinc finger-type factors	*Saccharomyces cerevisiae*
					HAP2 (MA0313.1)	Heteromeric CCAAT-binding	*Saccharomyces cerevisiae*
EOG092D01MX	Motif 28	SWI5 (MA0402.1)	A.T hook factors	*Saccharomyces cerevisiae*	SWI5 (MA0402.1)	A.T Hook factors	*Saccharomyces cerevisiae*
		ACE2 (MA0267.1)	C2H2 zinc finger factors	*Saccharomyces cerevisiae*	ACE2 (MA0267.1)	C2H2 zinc finger factors	*Saccharomyces cerevisiae*
		hmx2 (MA0897.1)	Homeo domain factors	*Mus musculus*			
EOG092D01WX	Motif 1	-	-	-	-	-	-
	Motif 2	-	-	-	-	-	-
	Motif 4				RPH1 (MA0372.1)	C2H2 zinc finger factors	*Saccharomyces cerevisiae*
					NSI1 (MA0421.1)	Tryptophan cluster factor	*Saccharomyces cerevisiae*
	Motif 6	-	-	-	-	-	-
	Motif 7	ATG669940 (MA1267.1)	C2H2 zinc finger factors	*Arabidopsis thaliana*	AZF1 (MA0277.1)	C2H2 zinc finger factors	*Saccharomyces cerevisiae*
		AT5G02460 (MA1281.1)	C2H2 zinc finger factors	*Arabidopsis thaliana*	SFL1 (MA0377.1)	Heat shock factors	*Saccharomyces cerevisiae*
		AT1G69570 (MA1268.1)	C2H2 zinc finger factors	*Arabidopsis thaliana*	FKH1 (MA0296.1)	Fork head/winged helix factors	*Saccharomyces cerevisiae*
		OBP3 (MA1274.1)	C2H2 zinc finger factors	*Arabidopsis thaliana*	HCM1 (MA0317.1)	Fork head/winged helix factors	*Saccharomyces cerevisiae*
		AT2G28810 (MA1272.1)	C2H2 zinc finger factors	*Arabidopsis thaliana*			
		OBP1 (MA1278.1)	C2H2 zinc finger factors	*Arabidopsis thaliana*			
		Adof1 (MA1277.1)	C2H2 zinc finger factors	*Arabidopsis thaliana*			
		COG1 (MA1279.1)	C2H2 zinc finger factors	*Arabidopsis thaliana*			
		JKD (MA1156.1)	C2H2 zinc finger factors	*Arabidopsis thaliana*			
		AT3G45610 (MA1270.1)	C2H2 zinc finger factors	*Arabidopsis thaliana*			
		MGP (MA1158.1)	C2H2 zinc finger factors	*Arabidopsis thaliana*			
		AT1G14580 (MA1160.1)	C2H2 zinc finger factors	*Arabidopsis thaliana*			
		NUC (MA1157.1)	C2H2 zinc finger factors	*Arabidopsis thaliana*			
		SGR5 (MA1159.1)	C2H2 zinc finger factors	*Arabidopsis thaliana*			
		AZF1 (MA0277.1)	C2H2 zinc finger factors	*Saccharomyces cerevisiae*			

		IDD4 (MA1371.1)	C2H2 zinc finger factors	*Arabidopsis thaliana*			
		IDD7 (MA1374.1)	C2H2 zinc finger factors	*Arabidopsis thaliana*			
		SOX10 (MA0442.2)	C2H2 zinc finger factors	*Arabidopsis thaliana*			
		IRF1 (MA0050.2)	Tryptophan cluster factors	*Arabidopsis thaliana*			
		Foxj3 (MA0851.1)	Fork head/winged helix factors	*Mus musculus*			
		ZNF384 (MA1125.1)	C2H2 zinc finger factors	*Homo sapiens*			
		SFL1 (MA0377.1)	Heat shock factors	*Saccharomyces cerevisiae*			
		IDD5 (MA1370.1)	C2H2 zinc finger factors	*Arabidopsis thaliana*			
	Motif 9	AP1 (MA0940.1)	MADS box factors	*Arabidopsis thaliana*	AZF1 (MA0277.1)	C2H2 zinc finger factors	*Saccharomyces cerevisiae*
		PI (MA0559.1)	MADS box factors	*Arabidopsis thaliana*	GCR2 (MA0305.1)		*Saccharomyces cerevisiae*
		AGL27 (MA1012.1)	MADS box factors	*Arabidopsis thaliana*	MCM1 (MA0331.1)	MADS box factors	*Saccharomyces cerevisiae*
		SEP3 (MA0563.1)	MADS box factors	*Arabidopsis thaliana*			
		AP3 (MA0556.1)	MADS box factors	*Arabidopsis thaliana*			
		AGL16 (MA1199.1)	MADS box factors	*Arabidopsis thaliana*			
		SOC1 (MA0554.1)	MADS box factors	*Arabidopsis thaliana*			
		AGL63 (MA1203.1)	MADS box factors	*Arabidopsis thaliana*			
		Id1 (MA0120.1)	C2H2 zinc finger factors	*Arabidopsis thaliana*			
		AT3G52440 (MA1276.1)	C2H2 zinc finger factors	*Arabidopsis thaliana*			
		AGL15 (MA0548.1)	MADS box factors	*Arabidopsis thaliana*			
		DAG2 (MA1271.1)	C2H2 zinc finger factors	*Arabidopsis thaliana*			
		AGL25 (MA1200.1)	MADS box factors	*Arabidopsis thaliana*			
EOG092D03RC	Motif 6	STB3 (MA0390.1)		*Saccharomyces cerevisiae*	STB3 (MA0390.1)		*Saccharomyces cerevisiae*
		CTCF (MA0139.1)	C2H2 zinc finger factors	*Homo sapiens*	HAC1 (MA0310.1)	Basic leucine zipper factors (bZIP)	*Saccharomyces cerevisiae*
	Motif 9	SOX10 (MA0442.2)	High-mobility group (HMG) domain factors	*Homo sapiens*	SPT23 (MA0388.1)		*Saccharomyces cerevisiae*
		AT1G69570 (MA1268.1)	C2H2 zinc finger factors	*Arabidopsis thaliana*	AZF1 (MA0277.1)	C2H2 zinc finger factors	*Saccharomyces cerevisiae*
		PI (MA0559.1)	MADS box factors	*Arabidopsis thaliana*	HCM1 (MA0317.1)	Fork head/winged helix factors	*Saccharomyces cerevisiae*
		OBP3 (MA1274.1)	C2H2 zinc finger factors	*Arabidopsis thaliana*	FKH2 (MA0297.1)	Fork head/winged helix factors	*Saccharomyces cerevisiae*
		AT5G66940 (MA1267.1)	C2H2 zinc finger factors	*Arabidopsis thaliana*	CUP2 (MA0287.1)	Copper-first DNA-binding domain	*Saccharomyces cerevisiae*

		IRF1 (MA0050.2)	Tryptophan cluster factors	*Homo sapiens*				
		Adof1 (MA1277.1)	C2H2 zinc finger factors	*Arabidopsis thaliana*				
		Foxj3 (MA0851.1)	Fork head/winged helix factors	*Mus musculus*				
	Motif 14	OBP1 (MA1278.1)	C2H2 zinc finger factors	*Arabidopsis thaliana*				
		ZNF263 (MA0528.1)	C2H2 zinc finger factors	*Homo sapiens*				
EOG092D05X9	Motif 15					TYE7 (MA0409.1)	Basic leucine zipper factors (bZIP)	*Saccharomyces cerevisiae*
						SWI4 (MA0401.1)	APSES-type DNA-binding domain	*Saccharomyces cerevisiae*
	Motif 1	E2F3 (MA0469.1)	Fork head/winged helix factors	*Homo sapiens*		YGR067C (MA0425.1)	C2H2 zinc finger factors	*Saccharomyces cerevisiae*
		E2F2 (MA0864.1)	Fork head/winged helix factors	*Homo sapiens*		AZF1 (MA0277.1)	C2H2 zinc finger factors	*Saccharomyces cerevisiae*
		SOX10 (MA0442.2)	C2H2 zinc finger factors	*Arabidopsis thaliana*				
		IRF1 (MA0050.2)	Tryptophan cluster factors	*Arabidopsis thaliana*				
		Foxj3 (MA0851.1)	Fork head/winged helix factors	*Mus musculus*				
		ZNF384 (MA1125.1)	C2H2 zinc finger factors	*Homo sapiens*				
		SFL1 (MA0377.1)	Heat shock factors	*Saccharomyces cerevisiae*				
		IDD5 (MA1370.1)	C2H2 zinc finger factors	*Arabidopsis thaliana*				
EOG092D0124	Motif 1	COG1 (MA1279.1)	C2H2 zinc finger factors	*Arabidopsis thaliana*		ROX1 (MA0371.1)	High-mobility group (HMG) domain factors	*Saccharomyces cerevisiae*
		OBP3 (MA1274.1)	C2H2 zinc finger factors	*Arabidopsis thaliana*				
		AT1G69570 (MA1268.1)	C2H2 zinc finger factors	*Arabidopsis thaliana*				
		AT5G66940 (MA1267.1)	C2H2 zinc finger factors	*Arabidopsis thaliana*				
		dof4.2 (MA1273.1)	C2H2 zinc finger factors	*Arabidopsis thaliana*				
		AT2G28810 (MA1272.1)	C2H2 zinc finger factors	*Arabidopsis thaliana*				
		FOXP2 (MA0593.1)	Fork head/winged helix factors	*Arabidopsis thaliana*				
		FOXP2 (MA0593.1)	Fork head/winged helix factors	*Arabidopsis thaliana*				
	Motif 12	At2g45680 (MA1285.1)	Basic leucine zipper (bZIP)	*Arabidopsis thaliana*				
	Motif 20	-	-	-		-	-	-
EOG092D0ACX	Motif 1	MA0576.1 (RAX3)	Tryptophan cluster factors	*Arabidopsis thaliana*		MA0381.1 (SKN7)	Heat shock factors	*Saccharomyces cerevisiae*
		MA1250.1 (AT1G75490)	AP2/ERF domain	*Arabidopsis thaliana*				

		MA0095.2 (YY1)	C2H2 zinc finger factors	*Homo sapiens*			
		MA1233.1 (AT1G71450)	AP2/ERF domain	*Arabidopsis thaliana*			
	Motif 2	MA0436.1 (YPR022C)	C2H2 zinc finger factors	*Saccharomyces cerevisiae*	MA0436.1 (YPR022C)	C2H2 zinc finger factors	*Saccharomyces cerevisiae*
		MA0732.1 (EGR3)	C2H2 zinc finger factors	*Saccharomyces cerevisiae*	MA0429.1 (YLL054C)	C6 zinc cluster factors	*Saccharomyces cerevisiae*
		MA0472.2 (EGR2)	C2H2 zinc finger factors	*Saccharomyces cerevisiae*	MA0339.1 (MIG3)	C2H2 zinc finger factors	*Saccharomyces cerevisiae*
					MA0338.1 (MIG2)	C2H2 zinc finger factors	*Saccharomyces cerevisiae*
	Motif 3	MA0403.1 (TBF1)	Tryptophan cluster factors	*Saccharomyces cerevisiae*	MA0403.1 (TBF1)	Tryptophan cluster factors	
		MA1073.1 (TRB2)	Tryptophan cluster factors	*Arabidopsis thaliana*	MA0372.1 (RPH1)	C2H2 zinc finger factors	
		MA0372.1 (RPH1)	C2H2 zinc finger factors	*Saccharomyces cerevisiae*			
	Motif 4	-	-	-	-	-	-
	Motif 5	MA1376.1 (DEAR3)	AP2/ERF domain	*Arabidopsis thaliana*			
		MA1368.1 (AT3G25990)	Helix-Turn-Helix	*Arabidopsis thaliana*			
	Motif 6	MA0337.1 (MIG1)	C2H2 zinc finger factors	*Saccharomyces cerevisiae*	MA0270.1 (AFT2)		*Saccharomyces cerevisiae*
		MA1410.1 (StBRC1)	Basic helix-loop-helix factors (bHLH)	*Solanum lycopersicum*	MA0337.1 (MIG1)	C2H2 zinc finger factors	*Saccharomyces cerevisiae*
		MA0270.1 (AFT2)		*Saccharomyces cerevisiae*	MA0441.1 (ZMS1)	C2H2 zinc finger factors	*Saccharomyces cerevisiae*
		MA0736.1 (GLIS2)	C2H2 zinc finger factors	*Homo sapiens*	MA0431.1 (TDA9)	C2H2 zinc finger factors	*Saccharomyces cerevisiae*
					MA0268.1 (ADR1)	C2H2 zinc finger factors	*Saccharomyces cerevisiae*
					MA0339.1 (MIG3)	C2H2 zinc finger factors	*Saccharomyces cerevisiae*
	Motif 8				MA0327.1 (HMRA1)	Homeo domain factors	*Saccharomyces cerevisiae*
	Motif 11	-	-	-	-	-	-
	Motif 18	-	-	-	-	-	-
	Motif 20	-	-	-	-	-	-
EOG092D0AI2	Motif 1	MA0281.1 (CBF1)	Basic helix-loop-helix factors (bHLH)	*Saccharomyces cerevisiae*	MA0281.1 (CBF1)	Basic helix-loop-helix factors (bHLH)	
					MA0325.1 (LYS14)	C6 zinc cluster factors	

					MA0384.1 (SNT2)	Tryptophan cluster factors	
					MA0409.1 (TYE7)	Basic helix-loop-helix factors (bHLH)	
	Motif 2	-	-	-	-	-	-
	Motif 6				MA0305.1 (GCR2)		*Saccharomyces cerevisiae*
					MA0285.1 (CRZ1)		*Saccharomyces cerevisiae*
					MA0304.1 (GCR1)		*Saccharomyces cerevisiae*
	Motif 7	MA0287.1 (CUP2)	Copper-fist DNA-binding domain	*Saccharomyces cerevisiae*	MA0287.1 (CUP2)	Copper-fist DNA-binding domain	*Saccharomyces cerevisiae*
		MA0402.1 (SWI5)	A.T hook factors	*Saccharomyces cerevisiae*	MA0402.1 (SWI5)	A.T hook factors	*Saccharomyces cerevisiae*
					MA0267.1 (ACE2)	C2H2 zinc finger factors	*Saccharomyces cerevisiae*
					MA0426.1 (YHP1)	Homeo domain factors	*Saccharomyces cerevisiae*
	Motif 8	MA1400.1 (At1g19000)	Helix-Turn-Helix	*Arabidopsis thaliana*			
	Motif 20	MA0865.1 (E2F8)	Fork head / winged helix factors	*Homo sapiens*			
		MA0758.1 (E2F7)	Fork head / winged helix factors	*Homo sapiens*			
EOG092D0564	Motif 2[A]	AT5G66940 (MA1267.1)	C2H2 zinc finger factors	*Arabidopsis thaliana*	RAP1 (MA0359.1)	Tryptophan cluster factor	*Saccharomyces cerevisiae*
		AT1G69570 (MA1268.1)	C2H2 zinc finger factors	*Arabidopsis thaliana*			
		ZNF24 (MA1124.1)	C2H2 zinc finger factors	*Arabidopsis thaliana*			
		At2g38090 (MA1193.1)	Helix-Turn-Helix	*Arabidopsis thaliana*			
		OBP1 (MA1278.1)	C2H2 zinc finger factors	*Arabidopsis thaliana*			
		AT5G02460 (MA1281.1)	C2H2 zinc finger factors	*Arabidopsis thaliana*			
	Motif 3[A]	AT1G69570 (MA1268.1)	C2H2 zinc finger factors	*Arabidopsis thaliana*	SFL1 (MA0377.1)	Heat shock factors	*Saccharomyces cerevisiae*
		OBP1 (MA1278.1)	C2H2 zinc finger factors	*Arabidopsis thaliana*	AZF1 (MA0277.1)	C2H2 zinc finger factors	*Saccharomyces cerevisiae*
		dof4.2 (MA1273.1)	C2H2 zinc finger factors	*Arabidopsis thaliana*	CUP2 (MA0287.1)	Copper-first DNA-binding domain	*Saccharomyces cerevisiae*
		AT5G66940 (MA1267.1)	C2H2 zinc finger factors	*Arabidopsis thaliana*			
		AT5G02460 (MA1281.1)	C2H2 zinc finger factors	*Arabidopsis thaliana*			
		AT2G28810 (MA1272.1)	C2H2 zinc finger factors	*Arabidopsis thaliana*			

		COG1 (MA1279.1)	C2H2 zinc finger factors	*Arabidopsis thaliana*			
		Adof1 (MA1277.1)	C2H2 zinc finger factors	*Arabidopsis thaliana*			
		OBP3 (MA1274.1)	C2H2 zinc finger factors	*Arabidopsis thaliana*			
		AT3G45610 (MA1270.1)	C2H2 zinc finger factors	*Arabidopsis thaliana*			
		SGR5 (MA1159.1)	C2H2 zinc finger factors	*Arabidopsis thaliana*			
		SVP (MA0555.1)	MADS box factors	*Arabidopsis thaliana*			
		JKD (MA1156.1)	C2H2 zinc finger factors	*Arabidopsis thaliana*			
		At2g38090 (MA1193.1)	Helix-Turn-Helix	*Arabidopsis thaliana*			
		IRF1 (MA0050.2)	Tryptophan cluster factors	*Arabidopsis thaliana*			
		MGP (MA1158.1)	C2H2 zinc finger factors	*Arabidopsis thaliana*			
		blmp-1 (MA0537.1)	C2H2 zinc finger factors	*Arabidopsis thaliana*			
		SOC1 (MA0554.1)	MADS box factors	*Arabidopsis thaliana*			
		OBP4 (MA1280.1)	C2H2 zinc finger factors	*Arabidopsis thaliana*			
		NFATC3 (MA0625.1)	Rel homology region (RHR) factors	*Homo sapiens*			
		AGL6 (MA1205.1)	MADS box factors	*Arabidopsis thaliana*			
		AT1G14580 (MA1160.1)	C2H2 zinc finger factors	*Arabidopsis thaliana*			
	Motif 4	-	-	-	-	-	-
EOG092D01IY	Motif 4[A]	OBP3 (MA127.1)	C2H2 zinc finger factors	*Arabidopsis thaliana*	AZF1 (MA0277.1)	C2H2 zinc finger factors	*Saccharomyces cerevisiae*
		AT1G69570 (MA1268.1)	C2H2 zinc finger factors	*Arabidopsis thaliana*	NHP6A (MA0345.1)	High-mobility group (HMG) domain factors	*Saccharomyces cerevisiae*
		OBP1 (MA1278.1)	C2H2 zinc finger factors	*Arabidopsis thaliana*			
		Adof1 (MA1277.1)	C2H2 zinc finger factors	*Arabidopsis thaliana*			
		AP1 (MA0940.1)	MADS box factors	*Arabidopsis thaliana*			
		AT2G28810 (MA1272.1)	C2H2 zinc finger factors	*Arabidopsis thaliana*			
		AT5G02460 (MA1281.1)	C2H2 zinc finger factors	*Arabidopsis thaliana*			
		COG1 (MA1279.1)	C2H2 zinc finger factors	*Arabidopsis thaliana*			
		AZF1 (MA0277.1)	C2H2 zinc finger factors	*Saccharomyces cerevisiae*			
		PI (MA0559)	MADS box factors	*Arabidopsis thaliana*			
		dof4.2 (MA1273.1)	C2H2 zinc finger factors	*Arabidopsis thaliana*			
		SOC1 (MA0554.1)	MADS box factors	*Arabidopsis thaliana*			

		SVP (MA0555.1)	MADS box factors	*Arabidopsis thaliana*			
		AT3G45610 (MA1270.1)	C2H2 zinc finger factors	*Arabidopsis thaliana*			
		FLC (MA0558.1)	MADS box factors	*Arabidopsis thaliana*			
	Motif 9[A]	AT5G66940 (MA1267.1)	C2H2 zinc finger factors	*Arabidopsis thaliana*	SFL1 (MA0377.1)	Heat shock factors	*Saccharomyces cerevisiae*
		AT1G69570 (MA1268.1)	C2H2 zinc finger factors	*Arabidopsis thaliana*	AZF1 (MA0277.1)	C2H2 zinc finger factors	*Saccharomyces cerevisiae*
		MGP (MA1158.1)	C2H2 zinc finger factors	*Arabidopsis thaliana*			
		JKD (MA1156.1)	C2H2 zinc finger factors	*Arabidopsis thaliana*			
		AT5G02460 (MA1281.1)	C2H2 zinc finger factors	*Arabidopsis thaliana*			
		AT2G28810 (MA1272.1)	C2H2 zinc finger factors	*Arabidopsis thaliana*			
		OBP3 (MA127.1)	C2H2 zinc finger factors	*Arabidopsis thaliana*			
		NUC (MA1157.1)	C2H2 zinc finger factors	*Arabidopsis thaliana*			
		AT1G14580 (MA1160.1)	C2H2 zinc finger factors	*Arabidopsis thaliana*			
		OBP1 (MA1278.1)	C2H2 zinc finger factors	*Arabidopsis thaliana*			
		SGR5 (MA1159.1)	C2H2 zinc finger factors	*Arabidopsis thaliana*			
		Adof1 (MA1277.1)	C2H2 zinc finger factors	*Arabidopsis thaliana*			
		AT1G76880 (MA1366.1)	Helix-Turn-Helix	*Arabidopsis thaliana*			
EOG092D01YA	Motif 1	-	-	-	-	-	-
	Motif 2	-	-	-	-	-	-
	Motif 3	-	-	-	-	-	-
	Motif 5	-	-	-	-	-	-
	Motif 8[A]	Adof1 (MA1277.1)	C2H2 zinc finger factors	*Arabidopsis thaliana*	GAT4 (MA0302.1)	Other C4 zinc finger-type factors	*Saccharomyces cerevisiae*
		AT5G66940 (MA1267.1)	C2H2 zinc finger factors	*Arabidopsis thaliana*			
		SGR5 (MA1159.1)	C2H2 zinc finger factors	*Arabidopsis thaliana*			
		AT2G28810 (MA1272.1)	C2H2 zinc finger factors	*Arabidopsis thaliana*			
		BPC5 (MA1403.1)	Other	*Arabidopsis thaliana*			
		OBP3 (MA1274.1)	C2H2 zinc finger factors	*Arabidopsis thaliana*			
		BPC1 (MA1404.1)	Other	*Arabidopsis thaliana*			
		AT5G02460 (MA1281.1)	C2H2 zinc finger factors	*Arabidopsis thaliana*			
		AT1G69570 (MA1268.1)	C2H2 zinc finger factors	*Arabidopsis thaliana*			

		dof4.2 (MA1273.1)	C2H2 zinc finger factors	*Arabidopsis thaliana*			
		COG1 (MA1279.1)	C2H2 zinc finger factors	*Arabidopsis thaliana*			
	Motif 9						
	Motif 18[A]	MA1277.1 (Adof1)	C2H2 zinc finger factors	*Arabidopsis thaliana*	MA0277.1 (AZF1)	C2H2 zinc finger factors	*Saccharomyces cerevisiae*
		MA1268.1 (AT1G69570)	C2H2 zinc finger factors	*Arabidopsis thaliana*	MA0377.1 (SFL1)	Heat shock factors	*Saccharomyces cerevisiae*
		MA1267.1 (AT5G66940)	C2H2 zinc finger factors	*Arabidopsis thaliana*			
		MA1274.1 (OBP3)	C2H2 zinc finger factors	*Arabidopsis thaliana*			
		MA1281.1 (AT5G02460)	C2H2 zinc finger factors	*Arabidopsis thaliana*			
		MA1272.1 (AT2G28810)	C2H2 zinc finger factors	*Arabidopsis thaliana*			
		MA1275.1 (AT1G47655)	C2H2 zinc finger factors	*Arabidopsis thaliana*			
		MA1270.1 (AT3G45610)	C2H2 zinc finger factors	*Arabidopsis thaliana*			
		MA1279.1 (COG1)	C2H2 zinc finger factors	*Arabidopsis thaliana*			
		MA1158.1 (MGP)	C2H2 zinc finger factors	*Arabidopsis thaliana*			
		MA1271.1 (DAG2)	C2H2 zinc finger factors	*Arabidopsis thaliana*			
		MA1160.1 (AT1G14580)	C2H2 zinc finger factors	*Arabidopsis thaliana*			
		MA1157.1 (NUC)	C2H2 zinc finger factors	*Arabidopsis thaliana*			
		MA1278.1 (OBP1)	C2H2 zinc finger factors	*Arabidopsis thaliana*			
		MA1156.1 (JKD)	C2H2 zinc finger factors	*Arabidopsis thaliana*			
		MA0277.1 (AZF1)	C2H2 zinc finger factors	*Saccharomyces cerevisiae*			
		MA1276.1 (AT3G52440)	C2H2 zinc finger factors	*Arabidopsis thaliana*			
		MA1371.1 (IDD4)	C2H2 zinc finger factors	*Arabidopsis thaliana*			
		MA1374.1 (IDD7)	C2H2 zinc finger factors	*Arabidopsis thaliana*			
		MA1370.1 (IDD5)	C2H2 zinc finger factors	*Arabidopsis thaliana*			
		MA0559.1 (PI)	MADS box factors	*Arabidopsis thaliana*			
		MA1366.1 (AT1G76880)	Helix-Turn-Helix	*Arabidopsis thaliana*			
		MA1280.1 (OBP4)	C2H2 zinc finger factors	*Arabidopsis thaliana*			
EOG092D0072	Motif 1[A]	ZNF263 (MA0528.1)	C2H2 zinc finger factors	*Homo sapiens*	FKH1 (MA0296.1)	Fork head/winged helix factors	*Saccharomyces cerevisiae*
		AT5G02460 (MA1281.1)	C2H2 zinc finger factors	*Arabidopsis thaliana*	YER130C (MA0423.1)	C2H2 zinc finger factors	*Saccharomyces cerevisiae*
		OBP1 (MA1278.1)	C2H2 zinc finger factors	*Arabidopsis thaliana*	FKH2 (MA0297.1)	Fork head/winged helix factors	*Saccharomyces cerevisiae*

	COG1 (MA1279.1)	C2H2 zinc finger factors	*Arabidopsis thaliana*	ROX1 (MA037.1)	High-mobility group (HMG) domain factors	*Saccharomyces cerevisiae*
	OBP3 (MA1274.1)	C2H2 zinc finger factors	*Arabidopsis thaliana*			
	AT1G69570 (MA1268.1)	C2H2 zinc finger factors	*Arabidopsis thaliana*			
	AT5G66940 (MA1267.1)	C2H2 zinc finger factors	*Arabidopsis thaliana*			
	dof4.2 (MA1273.1)	C2H2 zinc finger factors	*Arabidopsis thaliana*			
	AT2G28810 (MA1272.1)	C2H2 zinc finger factors	*Arabidopsis thaliana*			
	FOXP2 (MA0593.1)	Fork head/winged helix factors	*Homo sapiens*			
	FOXC2 (MA0846.1)	Fork head/winged helix factors	*Homo sapiens*			
Motif 4[A]	AT1G69570 (MA1268.1)	C2H2 zinc finger factors	*Arabidopsis thaliana*	AZF1 (MA0277.1)	C2H2 zinc finger factors	*Saccharomyces cerevisiae*
	AT5G66940 (MA1267.1)	C2H2 zinc finger factors	*Arabidopsis thaliana*	SFL1 (MA0377.1)	Heat shock factors	*Saccharomyces cerevisiae*
	OBP3 (MA1274.1)	C2H2 zinc finger factors	*Arabidopsis thaliana*			
	Adof1 (MA1277.1)	C2H2 zinc finger factors	*Arabidopsis thaliana*			
	AT5G02460 (MA1281.1)	C2H2 zinc finger factors	*Arabidopsis thaliana*			
	AZF1 (MA0277.1)	C2H2 zinc finger factors	*Saccharomyces cerevisiae*			
	COG1 (MA1279.1)	C2H2 zinc finger factors	*Arabidopsis thaliana*			
	AT2G28810 (MA1272.1)	C2H2 zinc finger factors	*Arabidopsis thaliana*			
	AT3G45610 (MA1270.1)	C2H2 zinc finger factors	*Arabidopsis thaliana*			
	PI (MA0559.1)	MADS box factors	*Arabidopsis thaliana*			
	dof4.2 (MA1273.1)	C2H2 zinc finger factors	*Arabidopsis thaliana*			
	OBP1 (MA1278.1)	C2H2 zinc finger factors	*Arabidopsis thaliana*			
	JDK (MA1156.1)	C2H2 zinc finger factors	*Arabidopsis thaliana*			
	MGP (MA1158.1)	C2H2 zinc finger factors	*Arabidopsis thaliana*			
	IRF1 (MA0050.2)	Tryptophan cluster factors	*Homo sapiens*			
	NUC (MA1157.1)	C2H2 zinc finger factors	*Arabidopsis thaliana*			
	BPC1 (MA1404.1)	Other	*Arabidopsis thaliana*			
	SGR5 (MA1159.1)	C2H2 zinc finger factors	*Arabidopsis thaliana*			
	AT1G14580 (MA1160.1)	C2H2 zinc finger factors	*Arabidopsis thaliana*			
	BPC5 (MA1403.1)	Other	*Arabidopsis thaliana*			

		blpp-1 (MA0537.1)	C2H2 zinc finger factors	*Arabidopsis thaliana*
		AT1G47655 (MA1275.1)	C2H2 zinc finger factors	*Arabidopsis thaliana*
		DOF5.3 (MA1071.1)	C2H2 zinc finger factors	*Arabidopsis thaliana*
		SOC1 (MA0554.1)	MADS box factors	*Arabidopsis thaliana*
		BPC6 (MA1402.1)	Other	*Arabidopsis thaliana*
EOG092D0454	Motif 22[A]	OBP3 (MA1274.1)	C2H2 zinc finger factors	*Arabidopsis thaliana*
		AT2G28810 (MA1272.1)	C2H2 zinc finger factors	*Arabidopsis thaliana*

[A] A-rich motifs

- No match to TF

Figure S1: The sequence logos of the A-rich motifs. The genes are represented by their BUSCO IDs.

A-rich motifs

EOG092D01IY

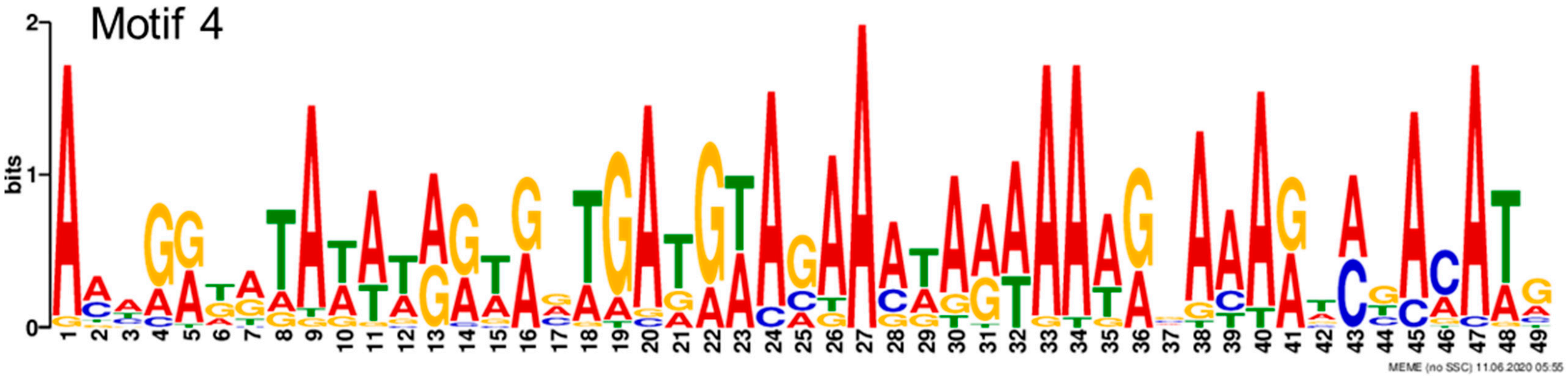

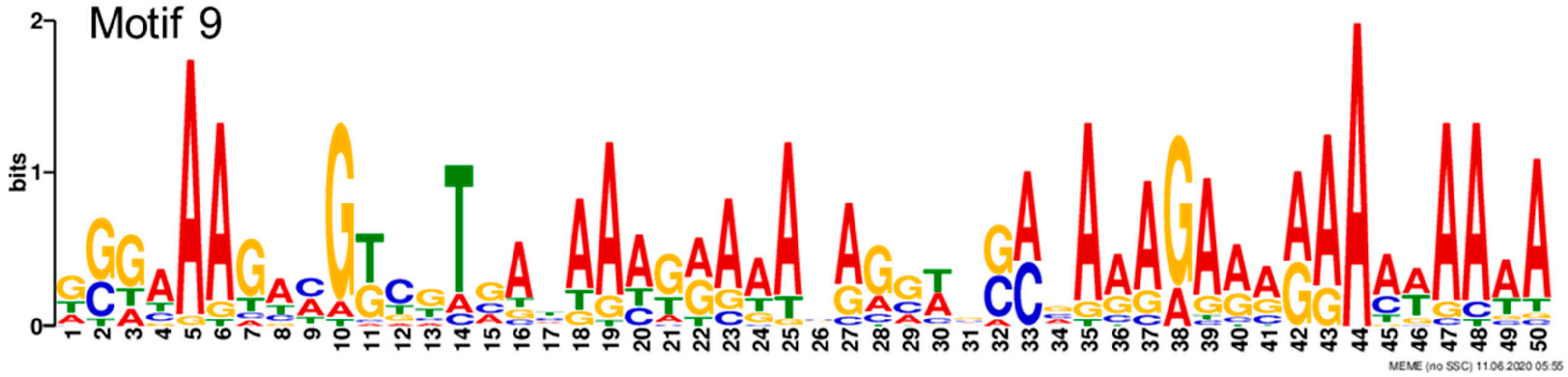

EOG092D0072

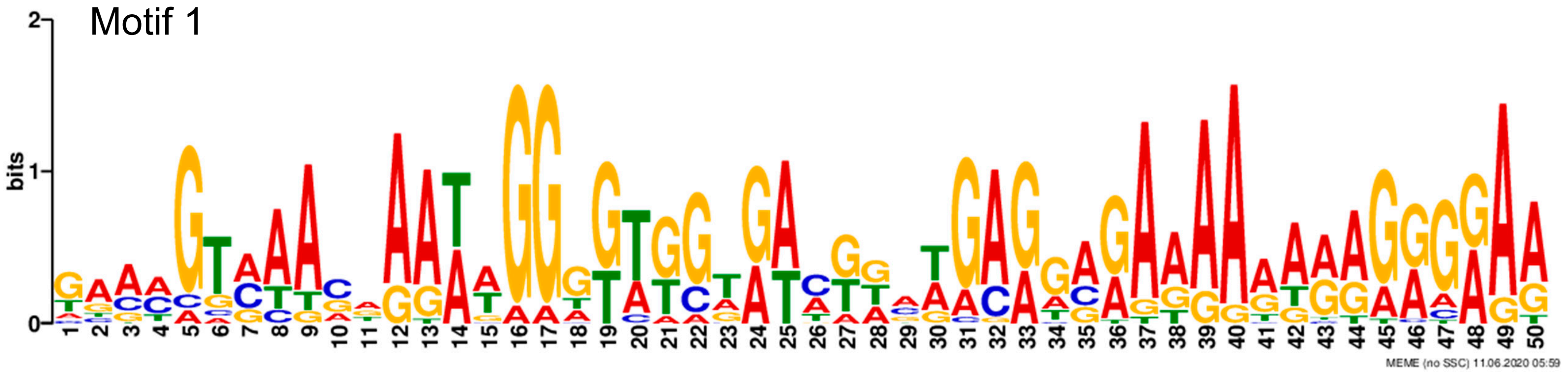

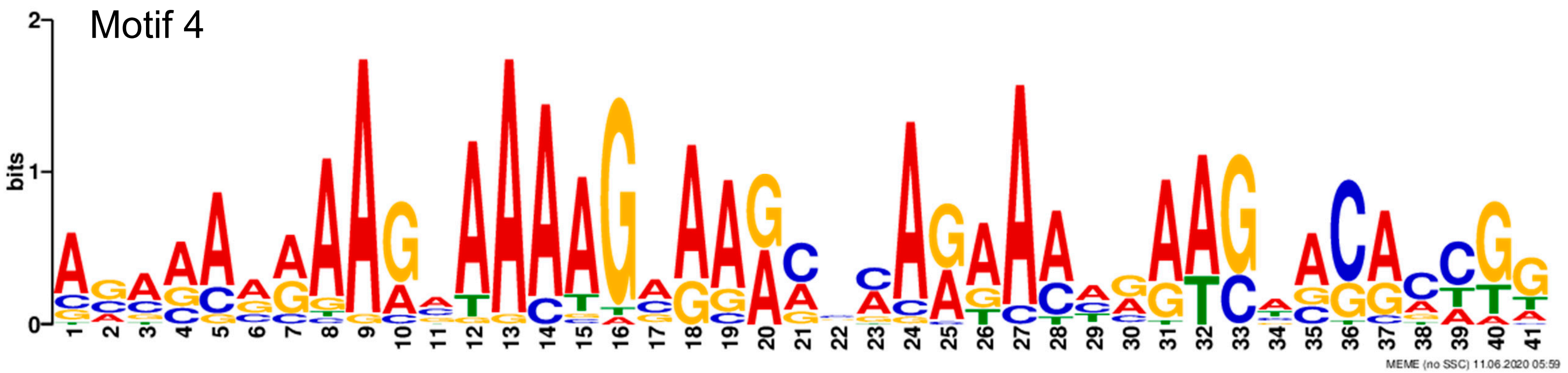

EOG092D01YA

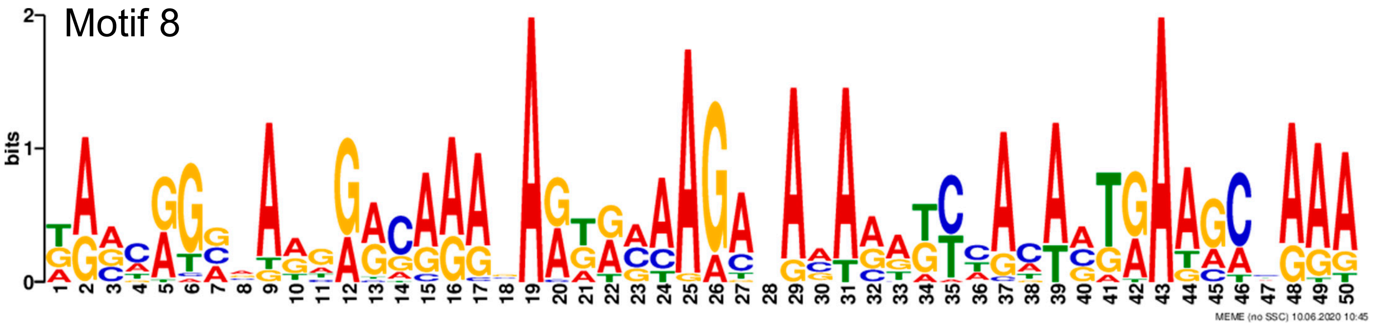

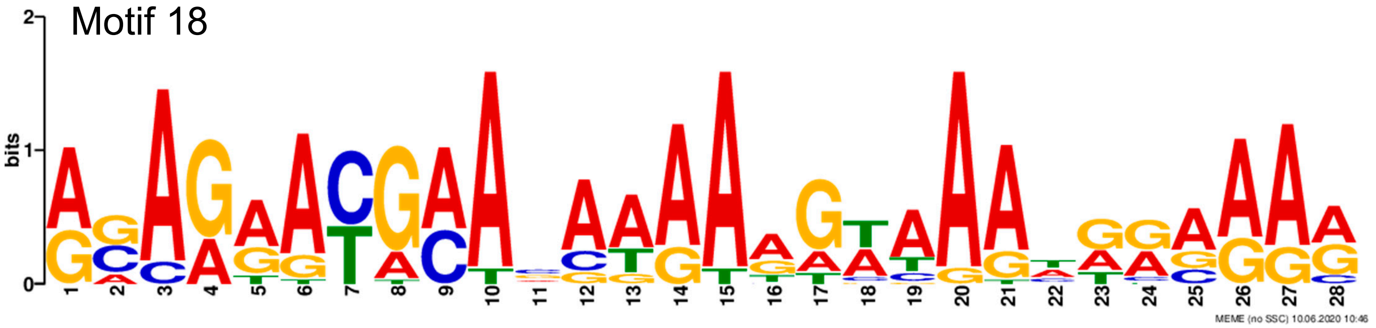

EOG092D0564

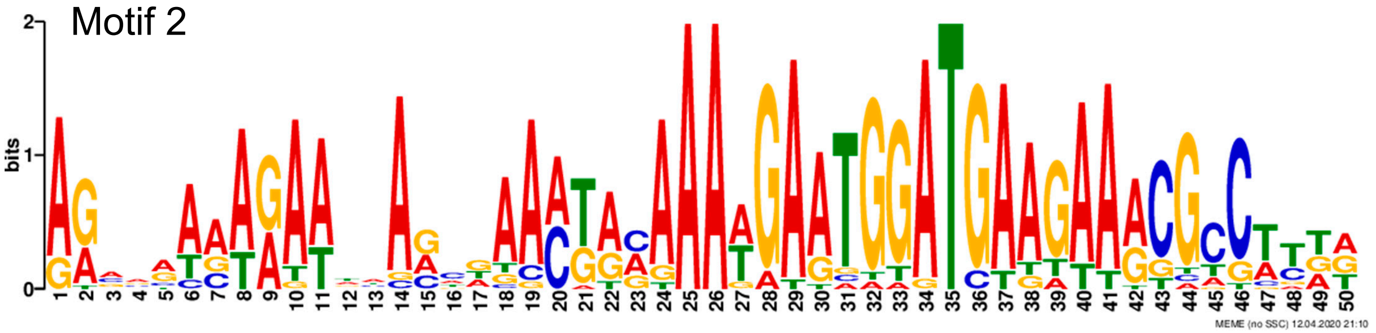

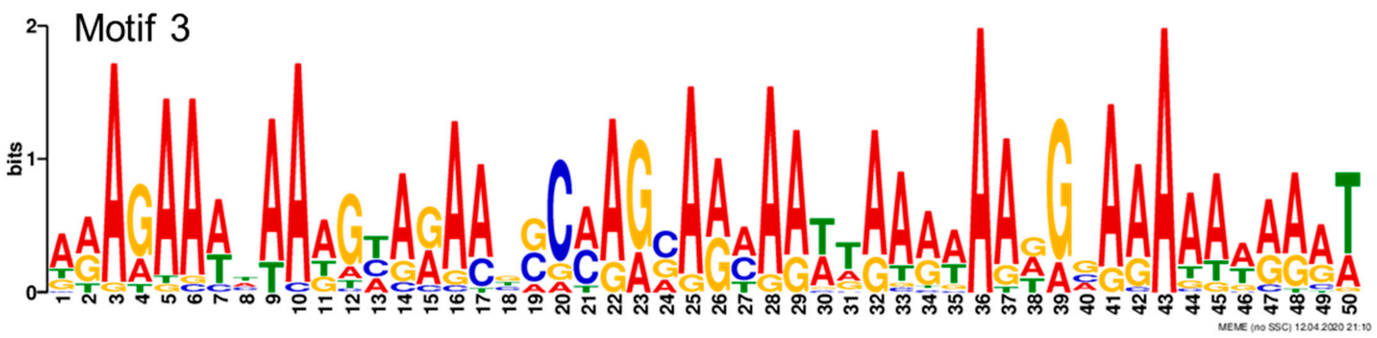

EOG092D0454

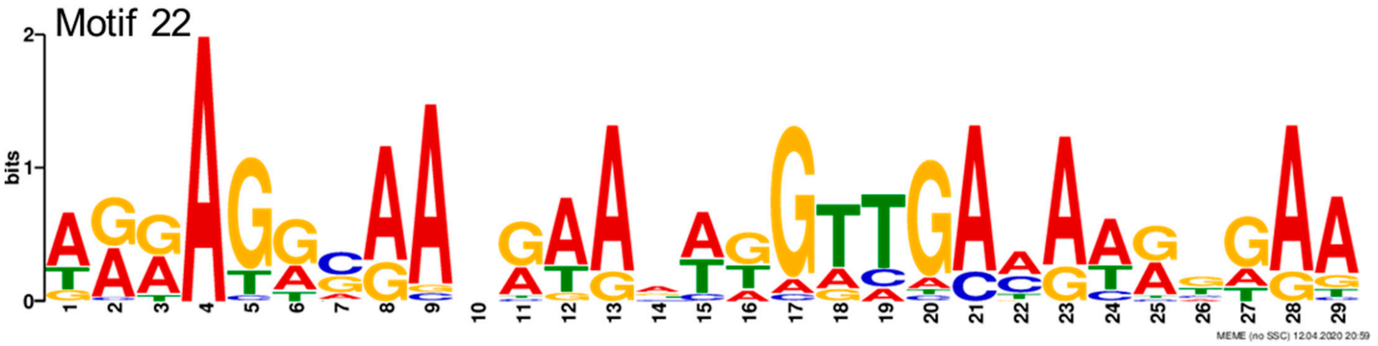

EOG092D01WX

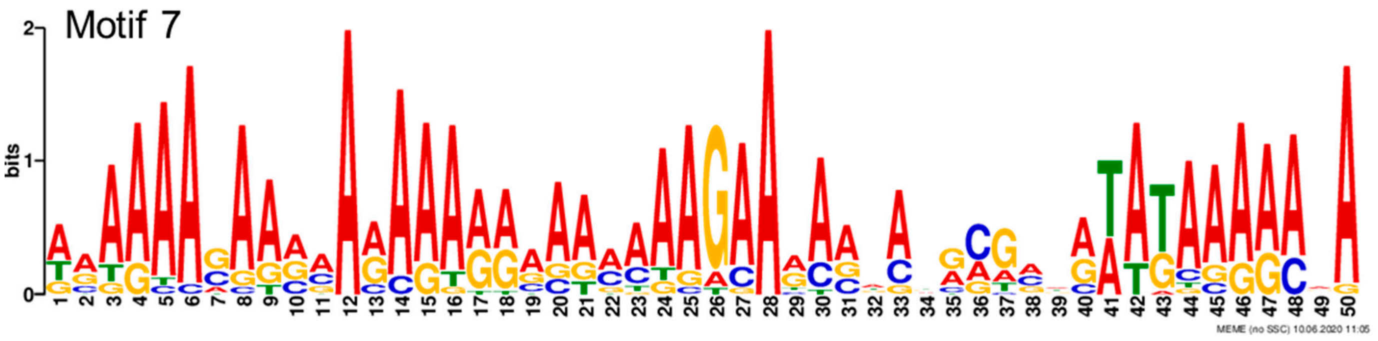

Figure S2: Positional distribution maps of motifs that met the cut-off requirements. The three different positional distribution patterns observed. The figures below show for each gene, the 1000 bp sequences used, the positions of each motif (each motif is represented by a different colour block, the height of the block is directly proportional to the E-value and the upside-down blocks represent motifs that are in reverse). These distribution maps were generated using the online tool MAST, which forms part of the MEME suite.

Pattern 1

EOG092D02YC

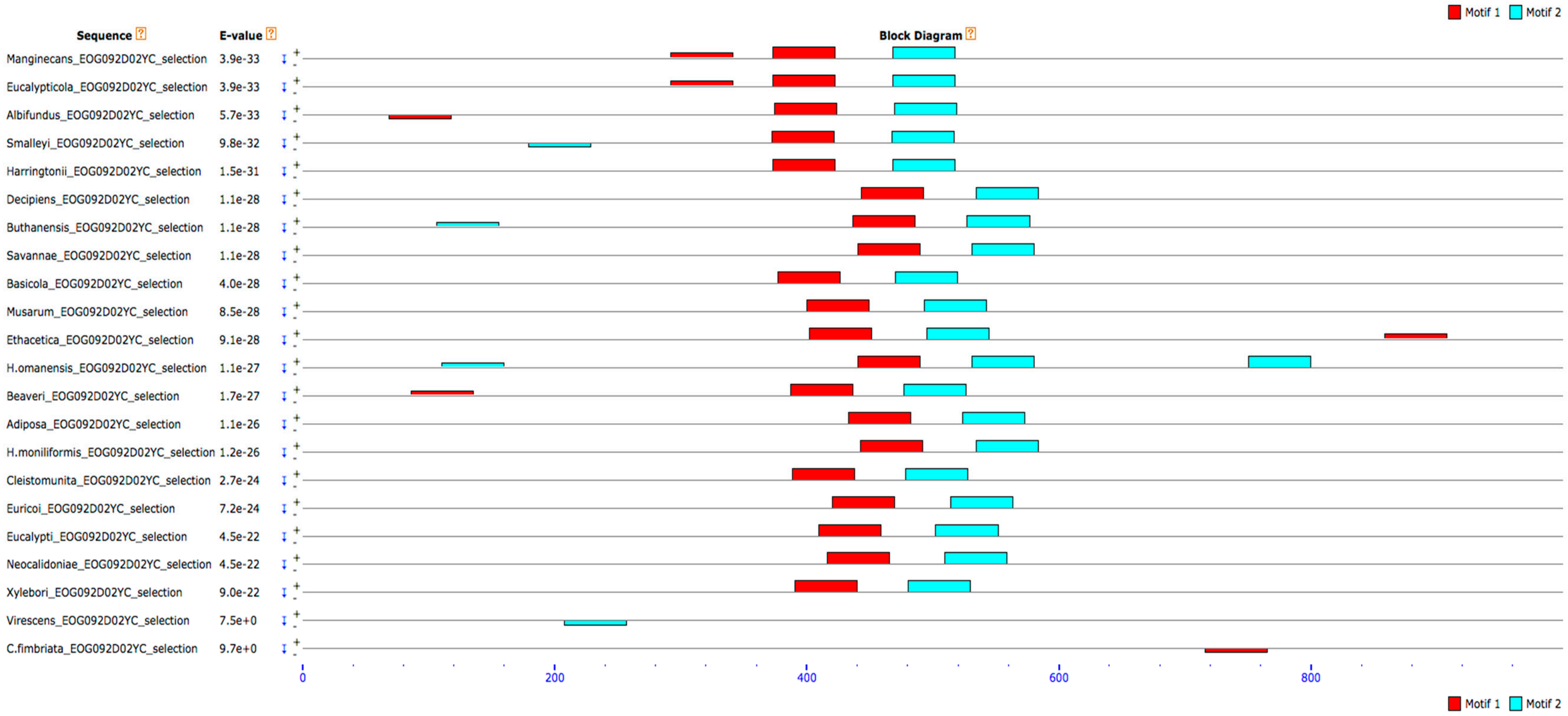

EOG092D00LL

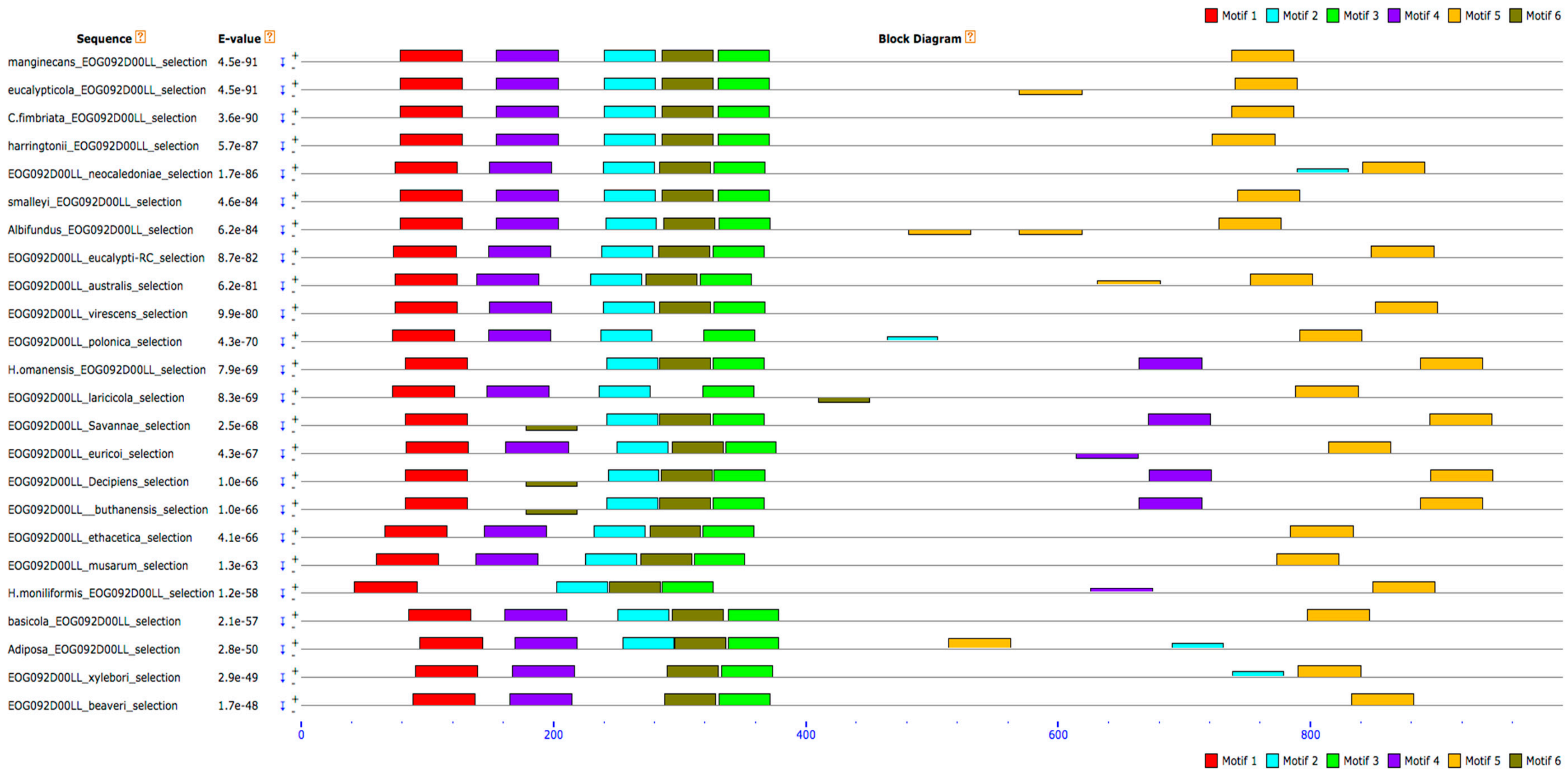

Pattern 2

EOG092D0072

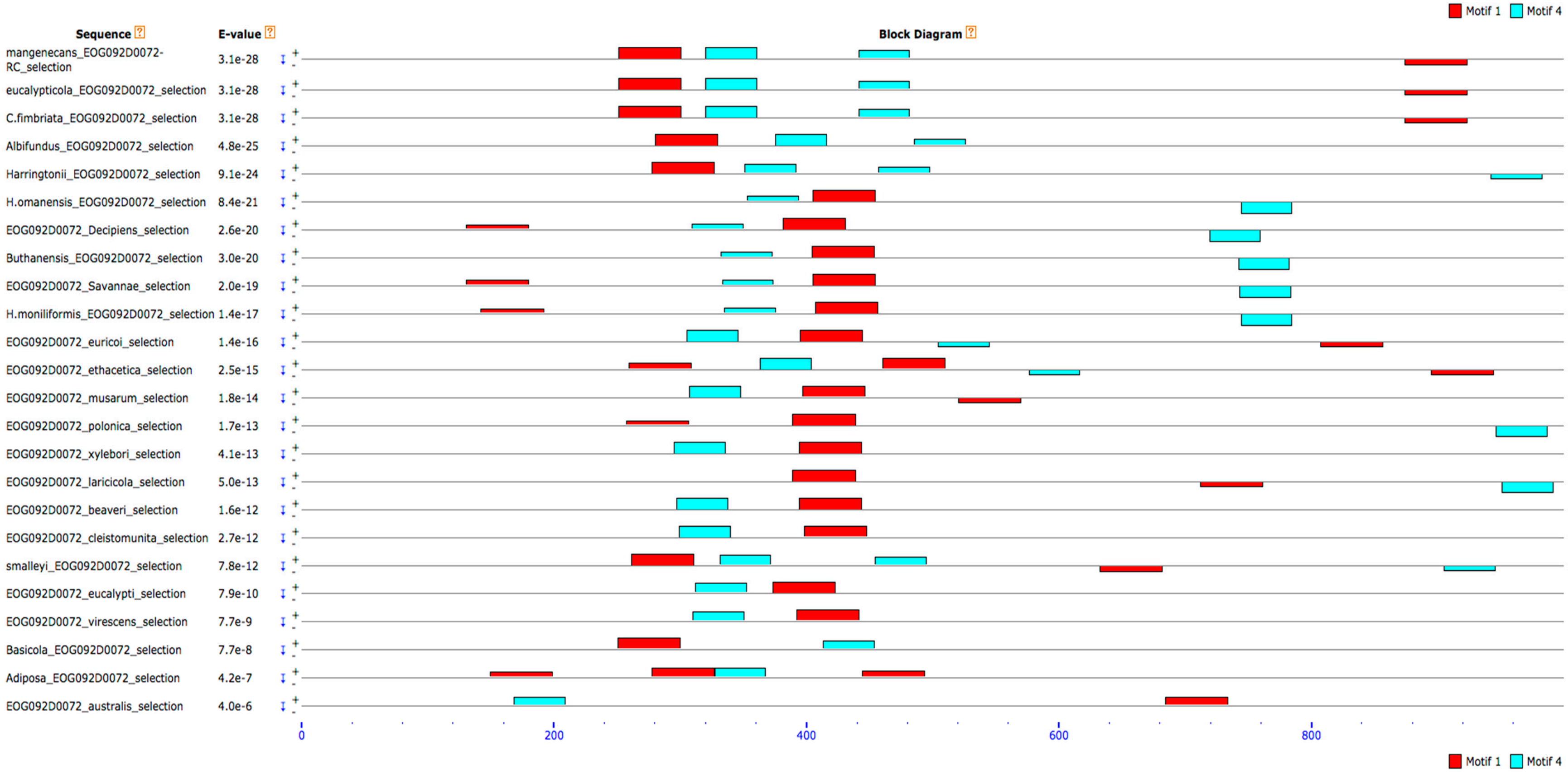

EOG092D01ZK

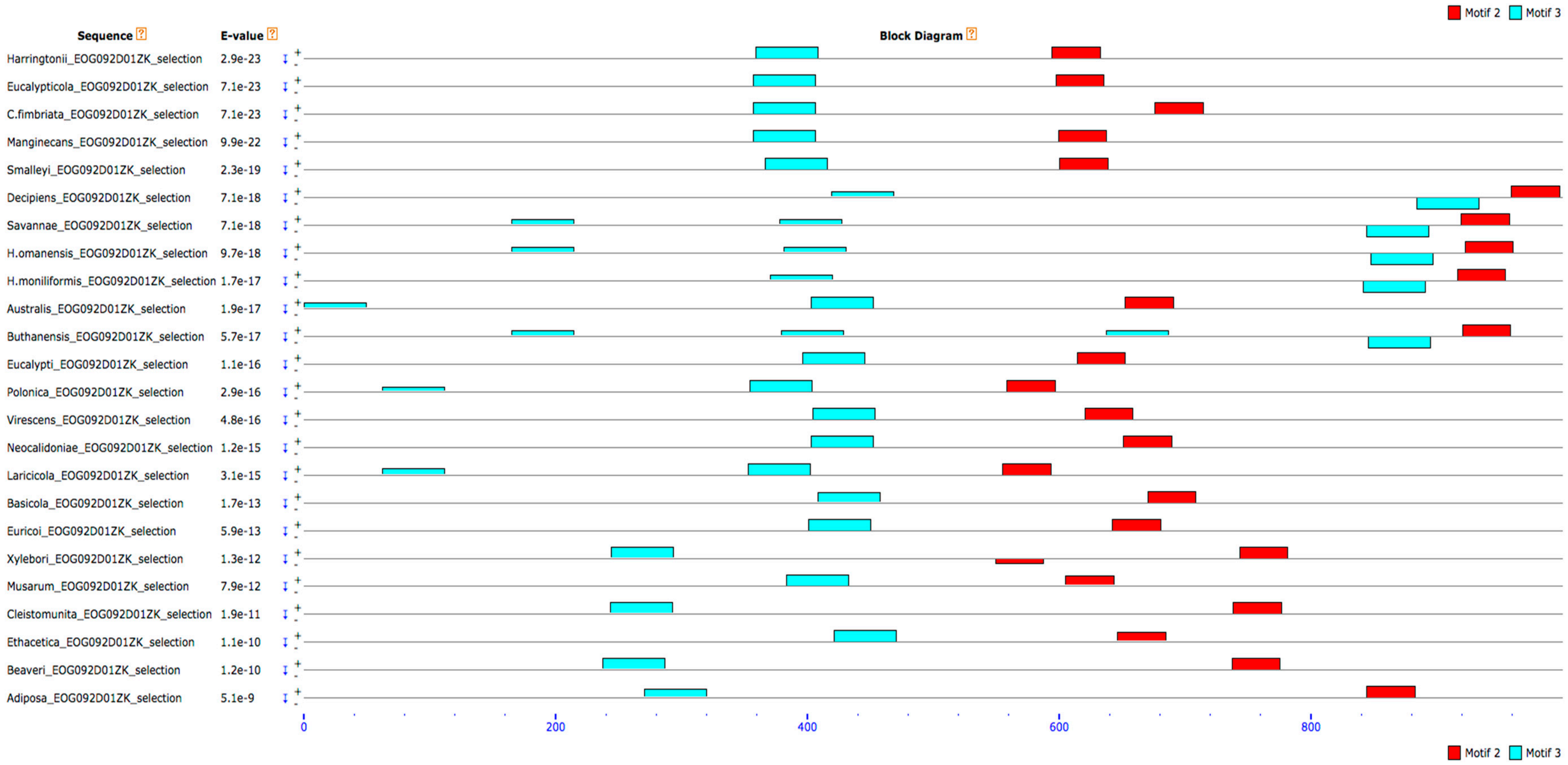

EOG092D01WX

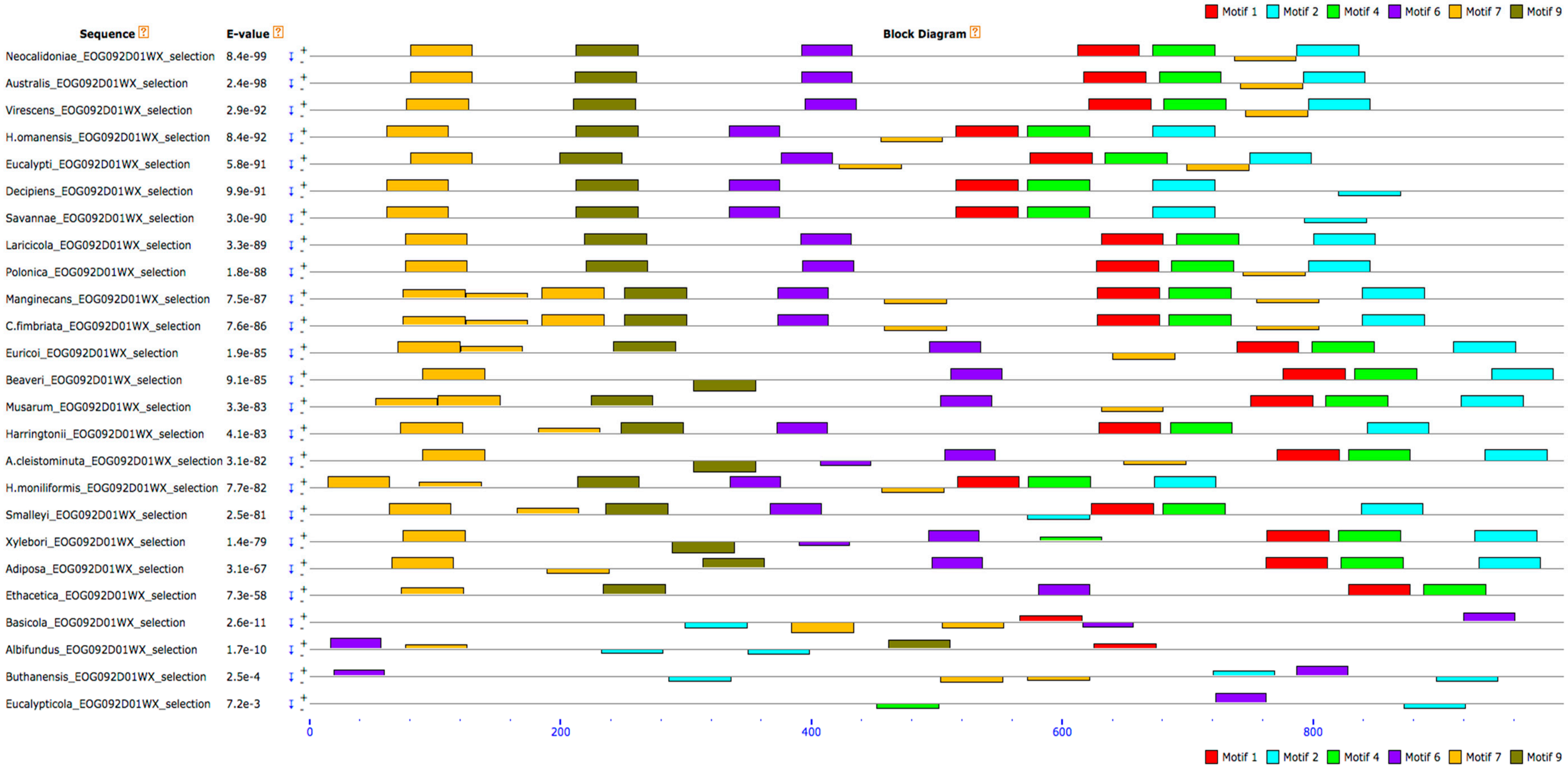

EOG092D01J4

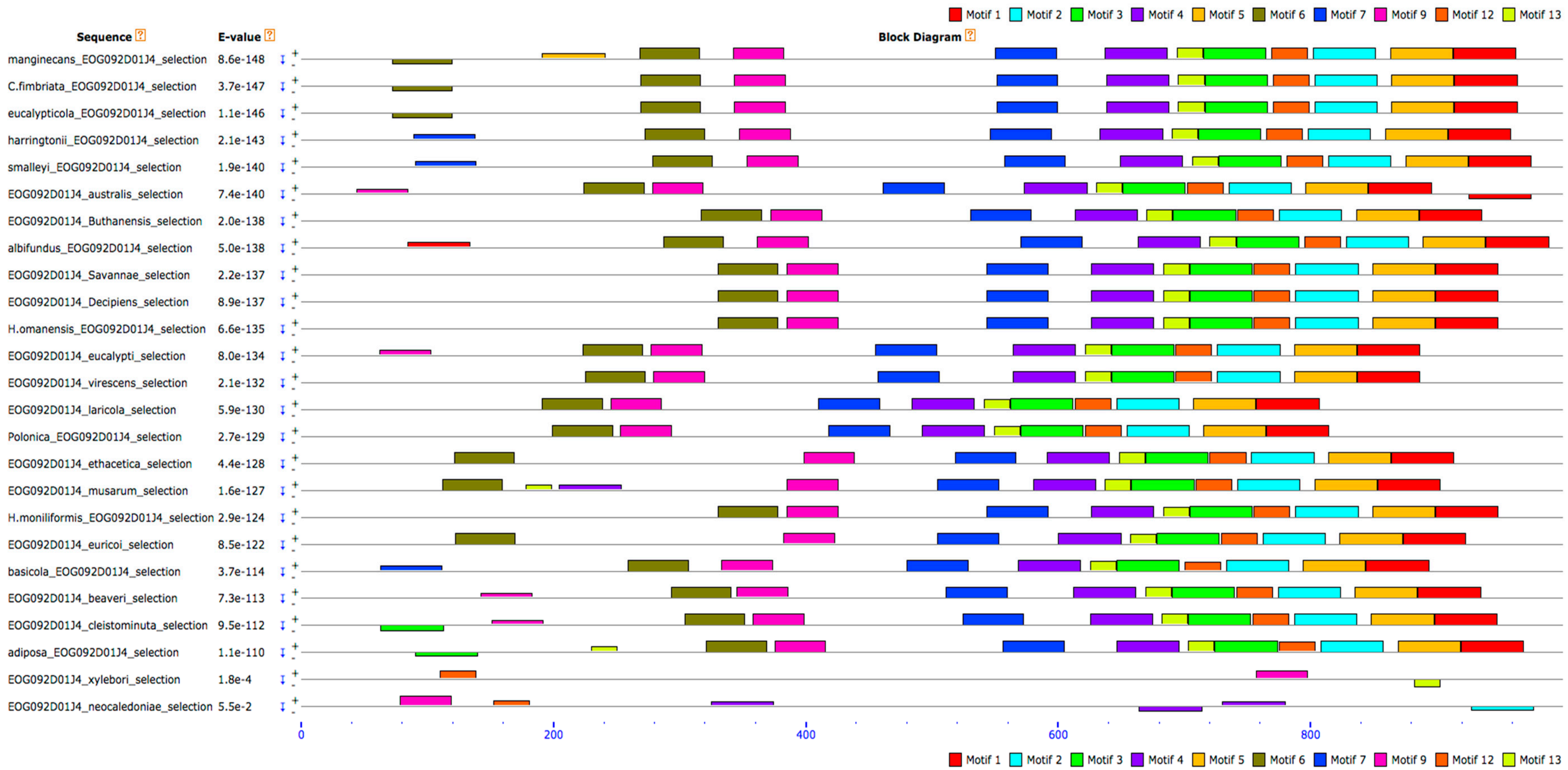

Pattern 3

EOG092D03RC

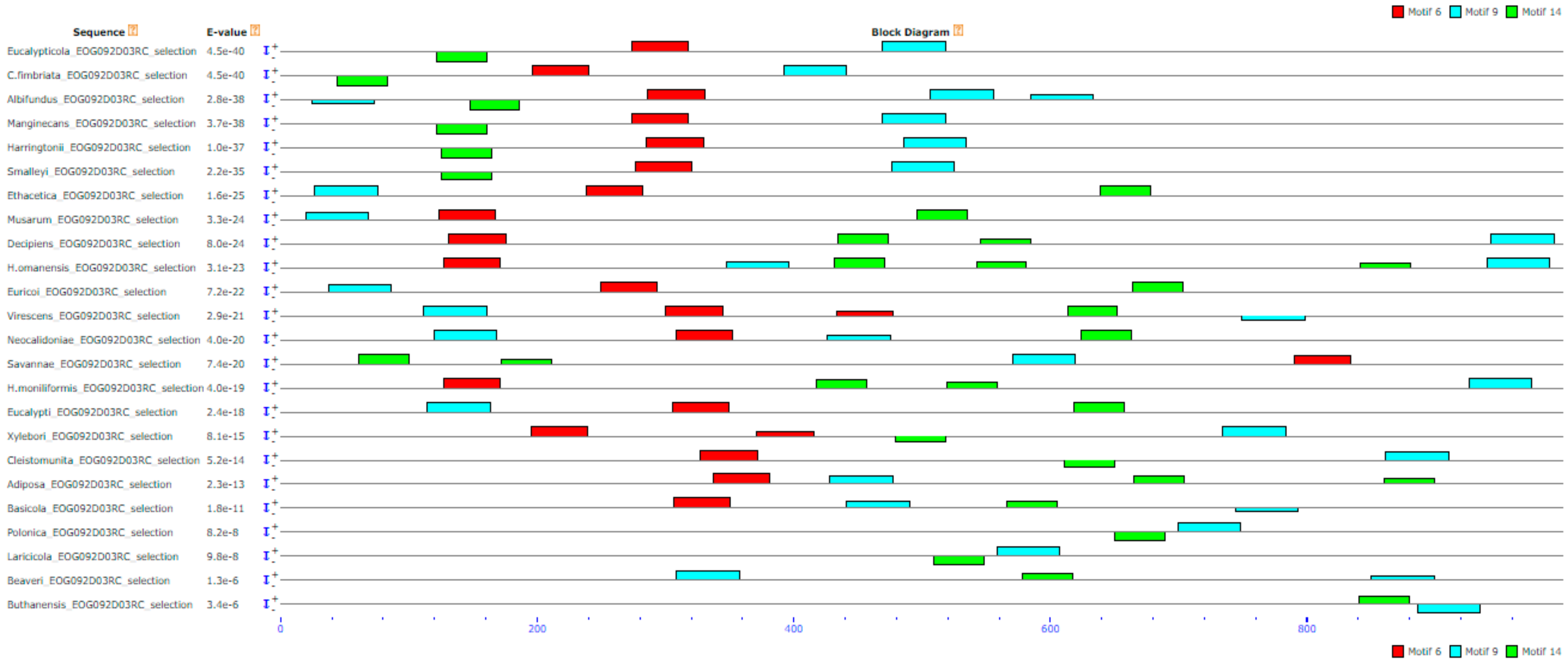

EOG092D0564

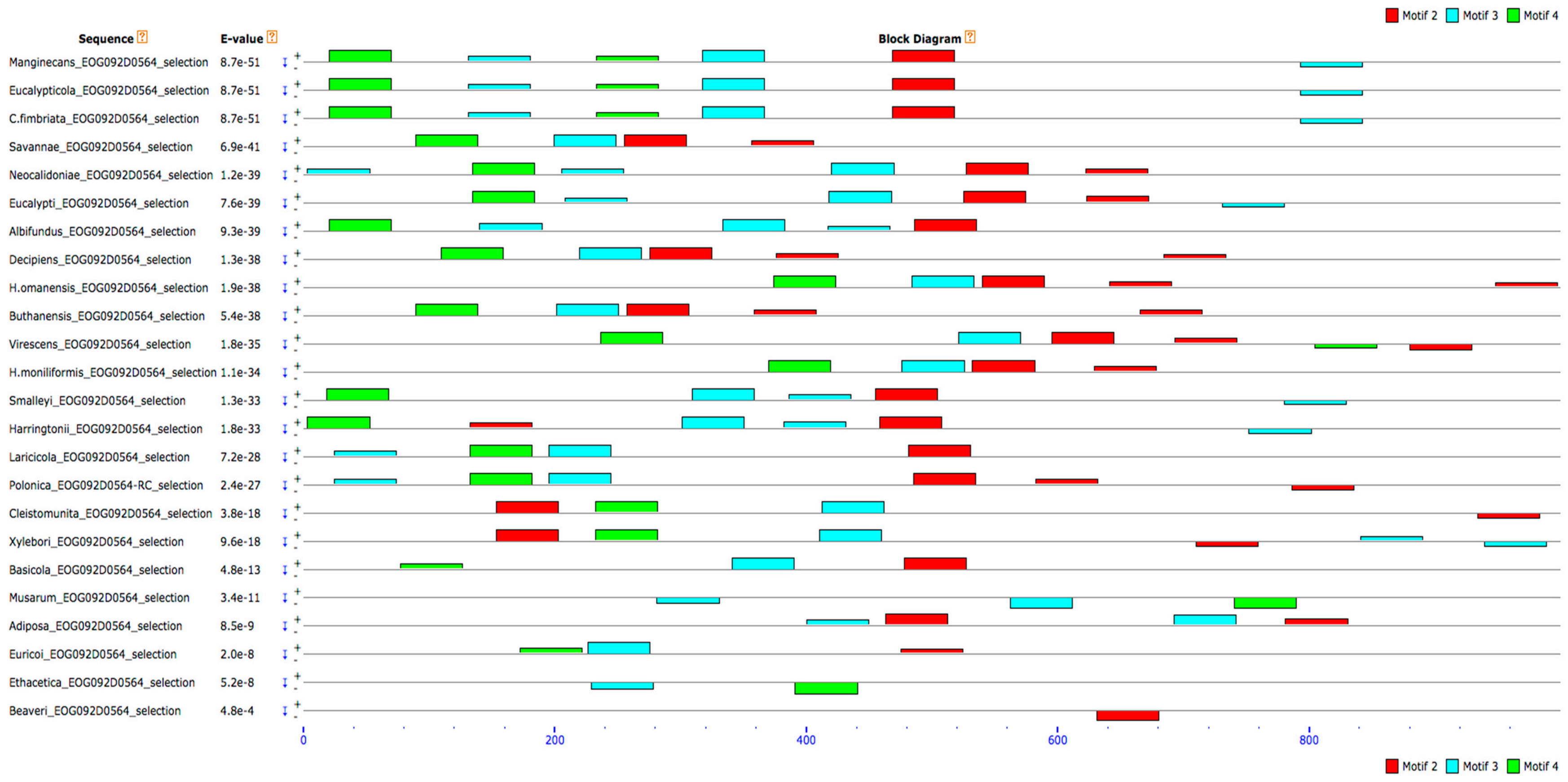

EOG092D0124

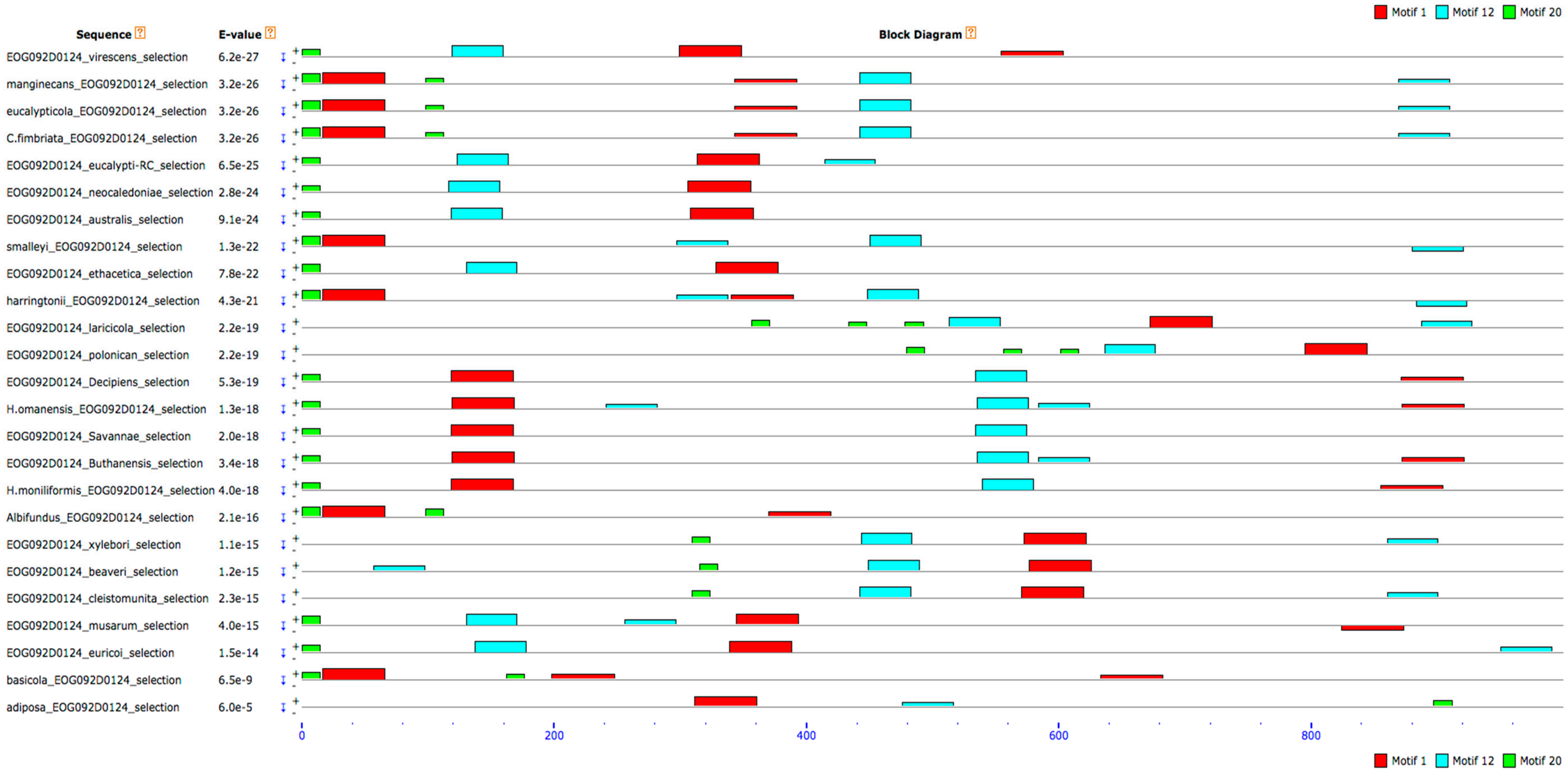

EOG092D05X9

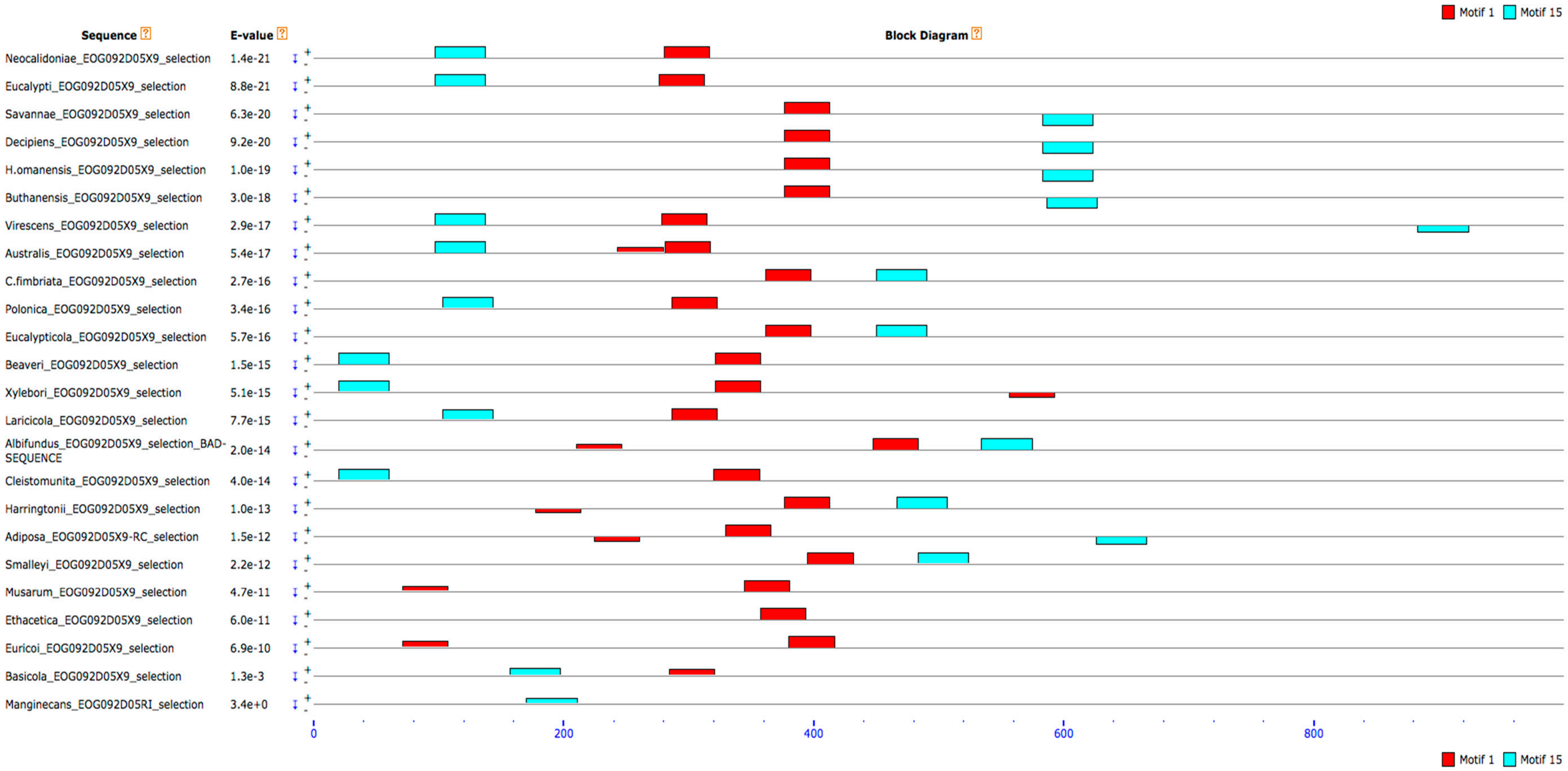

EOG092D03RY

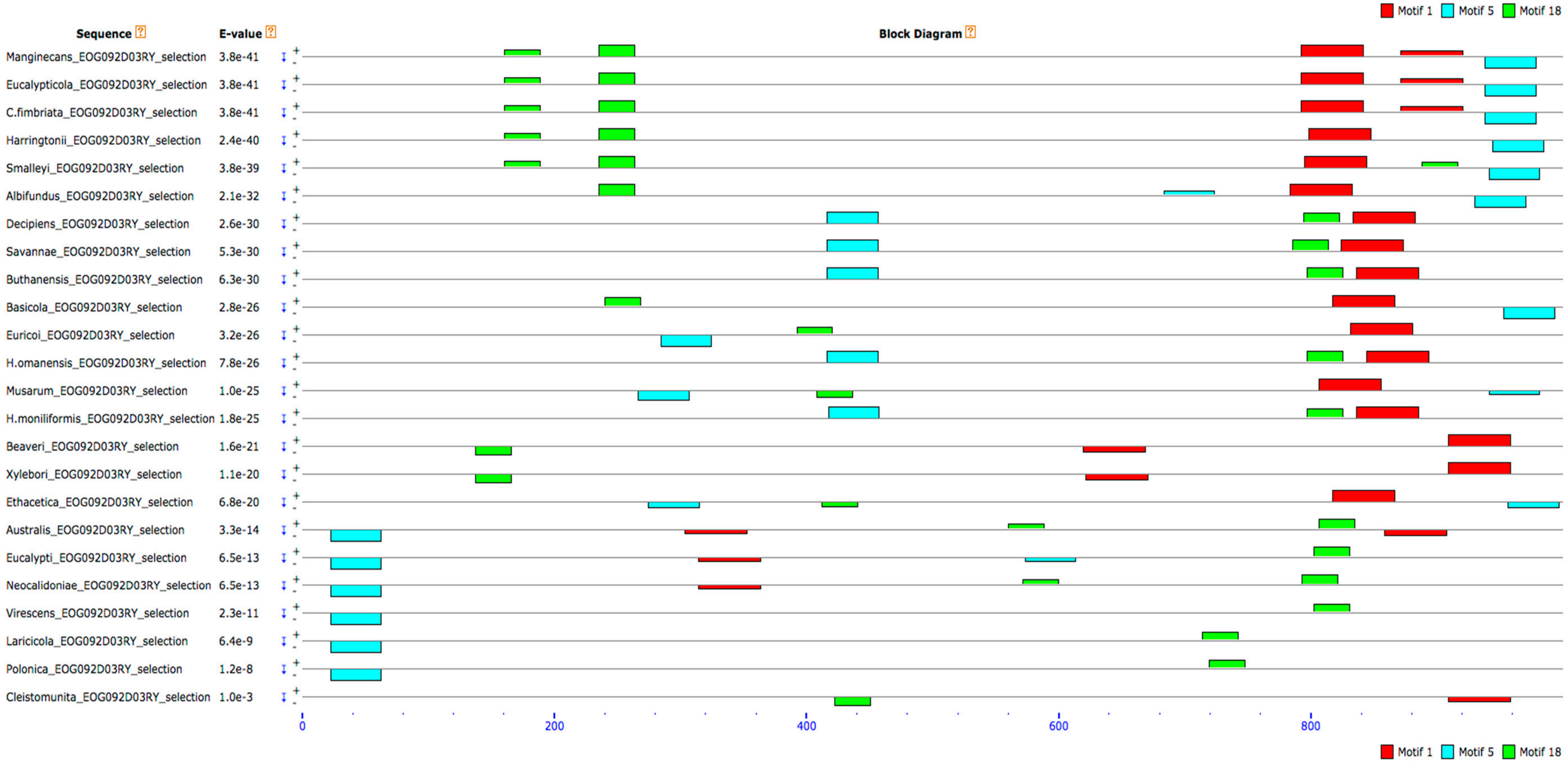

EOG092D01YA

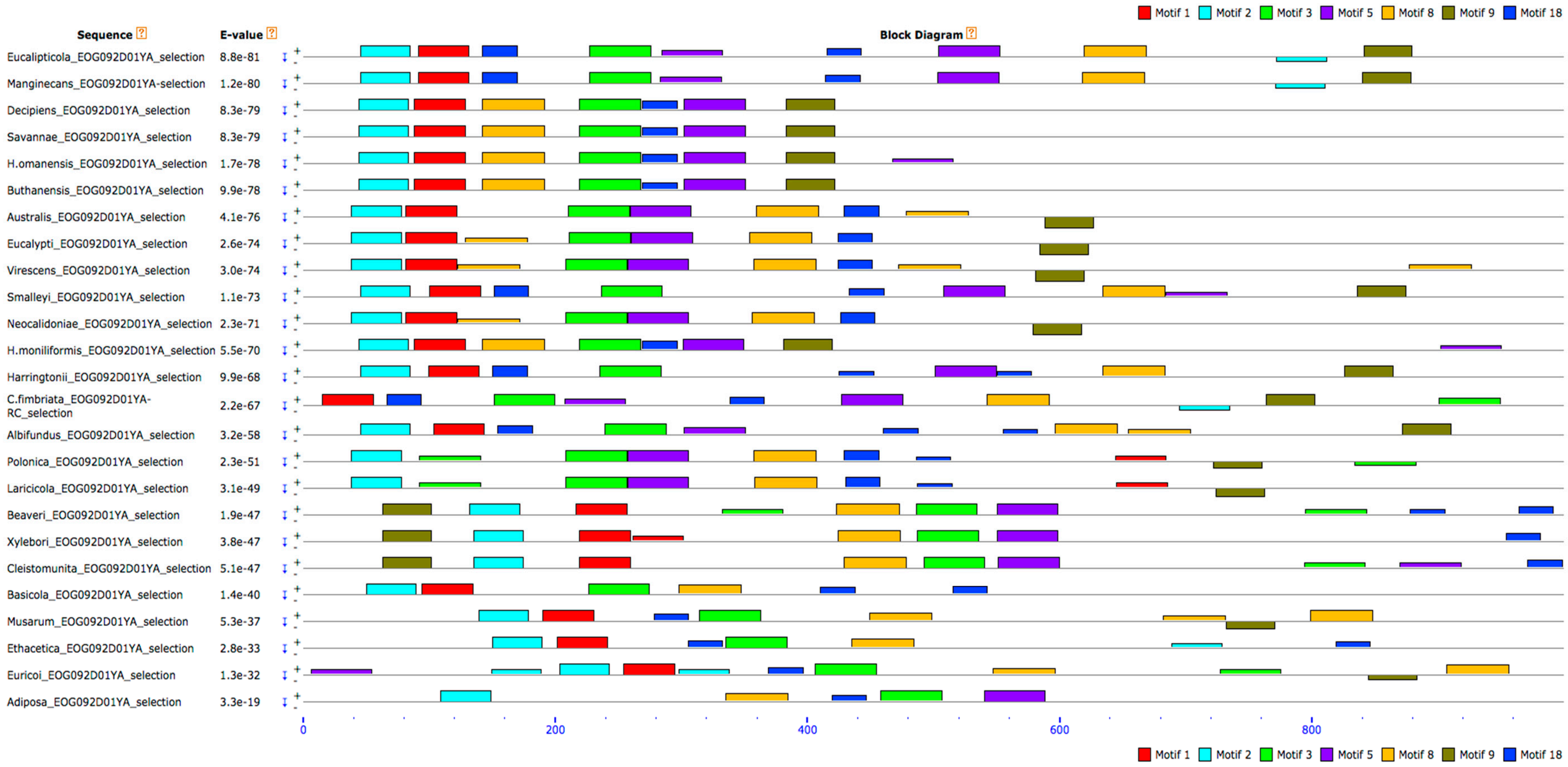

EOG092D01IY

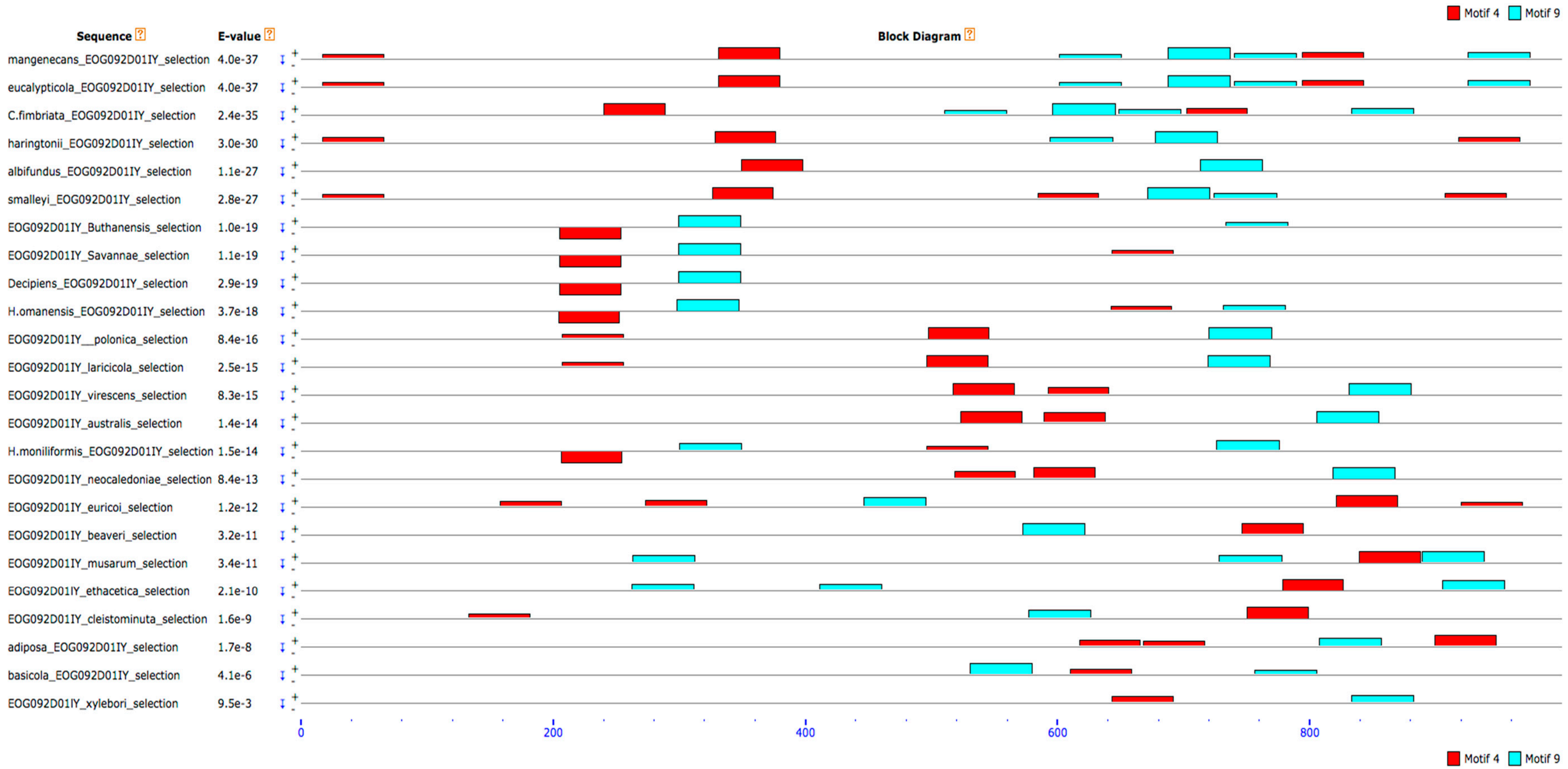

Only one motif

EOG092D01MX

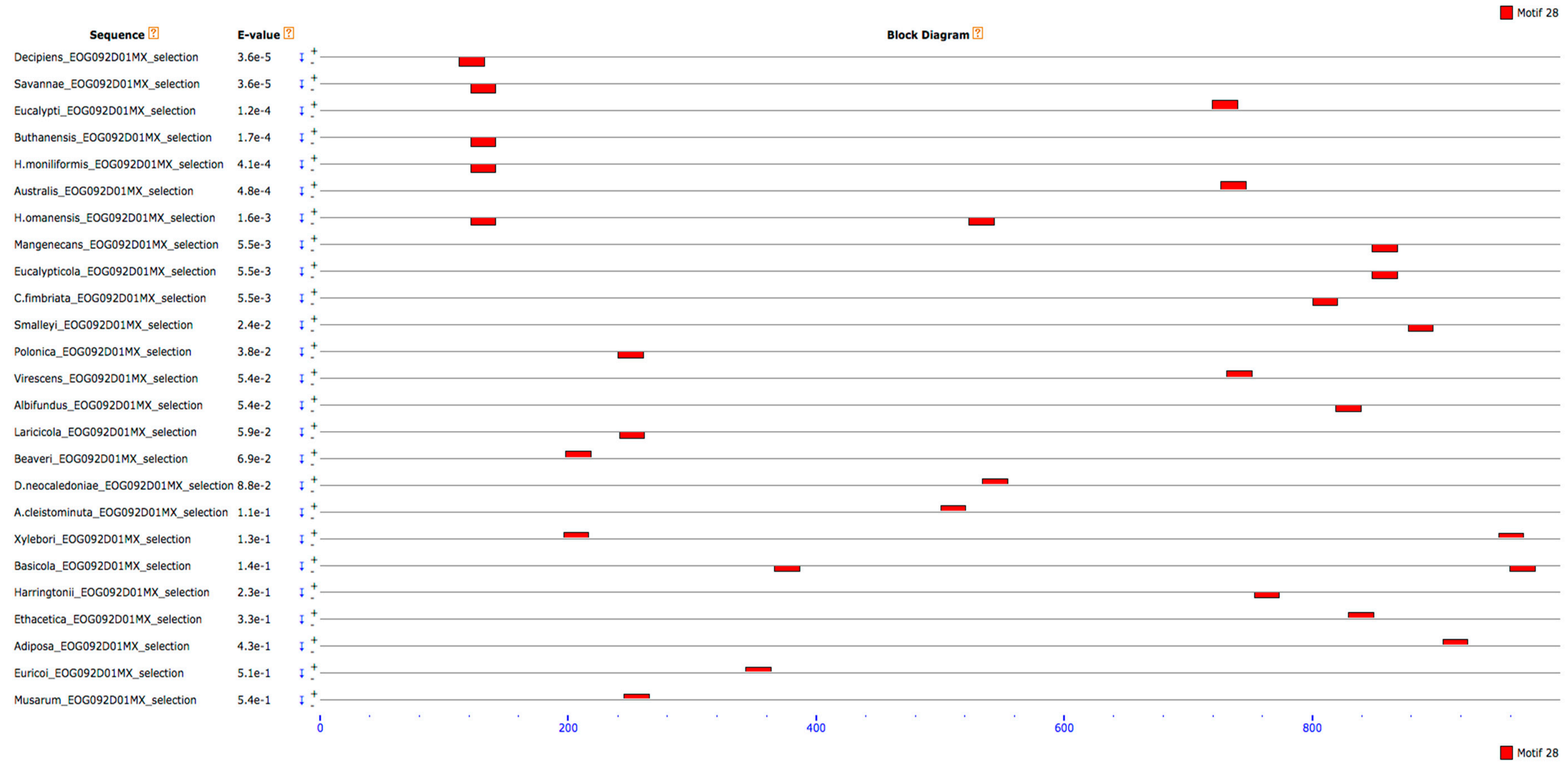

EOG092D0454

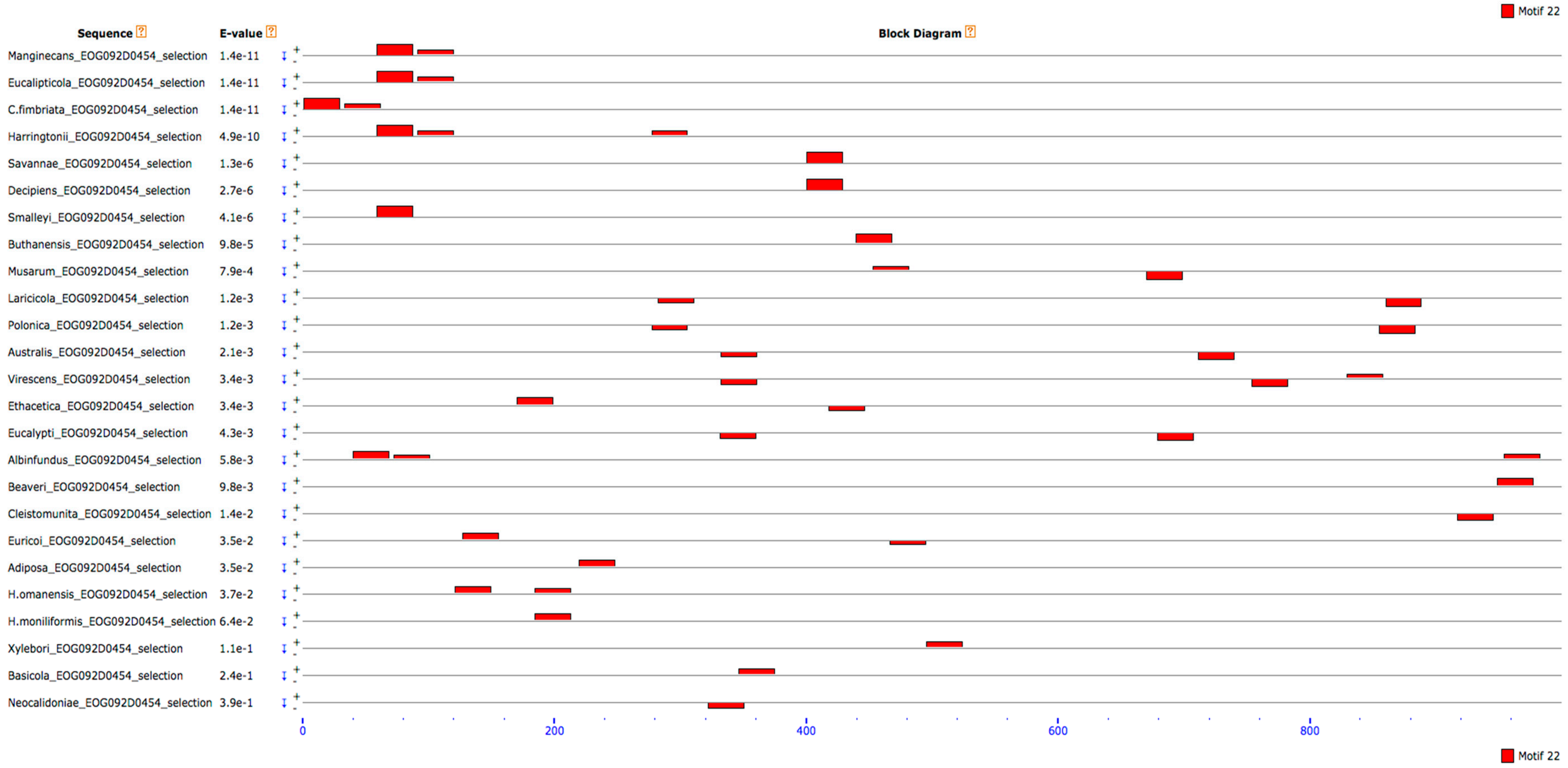

Supplement: Supplementary file 1 [file genes-14-00848-s001.zip › genes-2279659-supplementary.pdf]
